# Sleep Disturbances among Older Adults in the United States, 2002–2012: Nationwide Inpatient Rates, Predictors, and Outcomes

**DOI:** 10.3389/fnagi.2016.00266

**Published:** 2016-11-15

**Authors:** Alyssa A. Gamaldo, May A. Beydoun, Hind A. Beydoun, Hailun Liang, Rachel E. Salas, Alan B. Zonderman, Charlene E. Gamaldo, Shaker M. Eid

**Affiliations:** ^1^School of Aging Studies, University of South FloridaTampa, FL, USA; ^2^Behavioral Epidemiology Section, Laboratory of Epidemiology and Population Sciences, National Institute on Aging, NIA/NIH/IRPBaltimore, MD, USA; ^3^Human Development and Family Studies, Penn State UniversityState College, PA, USA; ^4^Department of Medicine, Johns Hopkins University School of MedicineBaltimore, MD, USA; ^5^Department of Health Policy and Management, Johns Hopkins University Bloomberg School of Public HealthBaltimore, MD, USA; ^6^Department of Neurology, Johns Hopkins University School of MedicineBaltimore, MD, USA

**Keywords:** sleep disturbances, sleep disorders, inpatient sample, co-morbidity, length of stay, health care cost, mortality, older adults

## Abstract

**Objective/Background:** We examined the rates, predictors, and outcomes [mortality risk (MR), length of stay (LOS), and total charges (TC)] of sleep disturbances in older hospitalized patients.

**Patients/Methods:** Using the U.S. Nationwide Inpatient Sample database (2002–2012), older patients (≥60 years) were selected and rates of insomnia, obstructive sleep apnea (OSA) and other sleep disturbances (OSD) were estimated using ICD-9CM. TC, adjusted for inflation, was of primary interest, while MR and LOS were secondary outcomes. Multivariable regression analyses were conducted.

**Results:** Of 35,258,031 older adults, 263,865 (0.75%) had insomnia, 750,851 (2.13%) OSA and 21,814 (0.06%) OSD. Insomnia rates increased significantly (0.27% in 2002 to 1.29 in 2012, *P*-trend < 0.001), with a similar trend observed for OSA (1.47 in 2006 to 5.01 in 2012, *P*-trend < 0.001). TC (2012 $) for insomnia-related hospital admission increased over time from $22,250 in 2002 to $31,527 in 2012, and increased similarly for OSA and OSD; while LOS and MR both decreased. Women with any sleep disturbance had lower MR and TC than men, while Whites had consistently higher odds of insomnia, OSA, and OSD than older Blacks and Hispanics. Co-morbidities such as depression, cardiovascular risk factors, and neurological disorders steadily increased over time in patients with sleep disturbances.

**Conclusion:** TC increased over time in patients with sleep disturbances while LOS and MR decreased. Further, research should focus on identifying the mechanisms that explain the association between increasing sleep disturbance rates and expenditures within hospital settings and the potential hospital expenditures of unrecognized sleep disturbances in the elderly.

## Introduction

Around half of community-dwelling older adults report sleep concerns (Neikrug and Ancoli-Israel, 2010), with overt sleep disorders frequently observed in this population (Wolkove et al., 2007; Neikrug and Ancoli-Israel, 2010; Roepke and Ancoli-Israel, 2010). Specifically, insomnia and obstructive sleep apnea (OSA) are two common disorders affecting Americans 60 years and older (Wolkove et al., 2007; Neikrug and Ancoli-Israel, 2010). The hospital, an under-studied setting, is pertinent since undetected or poorly treated sleep disturbances may account for poor prognosis and long-term utilization of hospital services, thereby increasing healthcare expenditures. As such, this study assesses trends of diagnosed sleep disturbances among older adults within the hospital setting and tests test whether these diagnoses rates are associated with hospitalization metrics (e.g., length of stay, mortality risk, and expenditures).

Sleep disturbances are typically comorbid with medical conditions (Gamaldo et al., 2012b), with the latter presenting obvious and unmanageable symptoms driving older adults to seek treatment in these settings. Those co-morbidities include heart disease (Budhiraja et al., 2011), hypertension (Budhiraja et al., 2011), diabetes (Gottlieb et al., 2005), stroke (Olafiranye et al., 2013), immune system dysfunction (Gamaldo et al., 2012b), stomach ulcers (Budhiraja et al., 2011), arthritis (Budhiraja et al., 2011), migraine (Budhiraja et al., 2011), asthma (Budhiraja et al., 2011), chronic obstructive pulmonary disease(COPD; Budhiraja et al., 2011), neurological complaints(Wolkove et al., 2007; Budhiraja et al., 2011), endocrine dysfunction (Budhiraja et al., 2011), depression and anxiety (Benca, 2014; Jaramillo et al., 2015). Severe levels of co-morbidity both threaten optimal patient care and constitute a public health and safety concern. Chronic untreated sleep disturbances are also linked to impaired cognitive functioning (Gamaldo et al., 2010, 2012a; Zimmerman and Aloia, 2012; Ford et al., 2015), increased traffic accidents (Terán-Santos et al., 1999; Budhiraja et al., 2011), worse quality of life (Daley et al., 2009; Ford et al., 2015), increased risk for disability (Daley et al., 2009; Ford et al., 2015), worse workplace productivity (Leger et al., 2012; Ford et al., 2015), and increased mortality (Hublin et al., 2011; Ford et al., 2015), exerting significant economic strain on the healthcare system (Peppard et al., 2013; Shear et al., 2014; Ford et al., 2015).

With population aging, healthcare service consumption will continuously increase amongst this demographic group at risk for developing and/or exacerbating ailments (Kaufmann et al., 2013). Evaluated as common in samples of community-dwelling older adults (Foley et al., 1995), sleep disturbances' rates remain largely unknown among older adult hospital inpatients. Exploring the hospital setting may support the need for healthcare providers to effectively recognize and treat sleep disturbances among older adults, especially if estimated sleep disturbance rates are lower than previously reported, suggesting under-reporting. Despite prior focus on the impact of sleep disturbances on older adults' physical and/or mental health (Wolkove et al., 2007; Gamaldo et al., 2010; Neikrug and Ancoli-Israel, 2010; Roepke and Ancoli-Israel, 2010), fewer studies examined the ramifications of older adults' sleep disturbances on their interface with the healthcare system particularly as it pertains to inpatient hospital stays. Limited research is available to understand sleep disturbance rates among older adults, examine trends over time, and assess whether co-morbidities and other individual-level and hospital-level characteristics are related to a sleep disturbance diagnosis (i.e., insomnia, OSA, or other sleep disturbance) and hospitalization outcomes.

The current study investigated five key objectives: **(A)** To assess over time trends in the rates of insomnia, OSA, and other sleep disturbances (OSD) among hospitalized older adults; **(B)** To compare patients with and without each of the three classes of sleep disturbance, in terms of co-morbidities, patient-level, and hospital-level characteristics in a recent period of time; **(C)** To compare hospitalization outcomes in patients with and without each of the three classes of sleep disturbance, mortality risk (MR), length of stay (LOS), and total charges (TC) in a recent period of time; **(D)** To examine trends in co-morbidity rates and outcomes of hospitalization among patients with each of the three classes of sleep disturbance; **(E)** To assess the predictive value of patient-level and hospital-level characteristics on outcomes of hospitalization among recently admitted patients with any of the three classes of sleep disturbance.

## Materials and methods

### Database and study participants

The Nationwide Inpatient Sample (NIS) is one of several databases and software tools implemented by the Healthcare Cost and Utilization Project (HCUP)^1^, a federal-state-industry partnership sponsored by the Agency for Healthcare Research and Quality (AHRQ). To date, the NIS is the largest all-payer hospital inpatient care database in the United States. Each year, NIS collects data on nearly 7–8 million hospital stays, reflecting discharges from ~1000 hospitals, a probability sample from HCUP State Inpatient Databases (SID). The sampling probability is ~20% and the design is stratified covering U.S. non-rehabilitation, community hospitals, with all acute care hospital discharges in the United States as the target universe. The NIS was developed to provide information on hospital utilization, charges, and quality of care in the United States

NIS defined its sampling strata using five hospital characteristics contained in the AHA hospital files: **(1)** Geographic Region—Northeast, Midwest, West, and South; **(2)** Control—public, private not-for-profit, and proprietary; **(3)** Location—urban or rural; **(4)** Teaching Status—teaching or non-teaching, **(5)** Bed Size—small, medium, and large.

The NIS includes clinical and resource-use information contained within a typical discharge abstract, protecting privacy of patients, physicians, and hospitals. Although NIS data are available since 1988, severity and comorbidity measures contained in the *severity* file became available from 2002 onwards. In addition, NIS did not add new states to its 35-state geographical coverage since 2002 providing more homogeneity in data acquisition over time. Therefore, we used NIS data from 2002 to 2012. Despite the redesign made in 2012, examining trends in means and proportions over the years is possible by inclusion of trend weights, allowing for comparable estimates for all years. (See Appendix I for more details).

### Diagnostic criteria

Each year, the *core* file of NIS provided the International Classification of Diseases, Ninth Revision, Clinical Modification (ICD-9-CM) codes for diagnosis and procedure, from discharge abstract, with information recoded retrospectively. The latest changes to ICD-9-CM codes are provided for 2011: http://www.cdc.gov/nchs/data/icd/ICD-9-CM%20TABULARADDENDAfy12.pdf. The number of diagnoses provided per patient varied by State. However, the number was truncated at 15 for both diagnoses and procedures because few cases had more than 15 diagnoses or procedures between 2002 and 2008. Nevertheless, two variables were provided indicating the total number of diagnoses and procedures. Between 2009 and 2012, up to 25 diagnoses were provided. However, for equal opportunity for a specific diagnosis, only the first 15 were considered for any year.

Among the possible 15 diagnoses, the first ranked diagnosis is termed “principal diagnosis.” In our main analysis, trends, characteristics, and outcomes of sleep disturbance as “any diagnosis” of 15 was of primary interest. ICD-9-CM codes used for each class of sleep disturbance as determined by clinical sleep specialist are outline in Appendix II.

### Co-morbidity measure

The AHRQ comorbidity measures identify coexisting medical conditions not directly related to principal diagnosis or main reason for admission, and are likely to have originated before hospital stay. The AHRQ comorbidity measures were developed as one of the HCUP tools. Complete documentation on the comorbidity measures is available on the HCUP User Support Website under Tools & Software. (http://www.hcup-us.ahrq.gov/toolssoftware/comorbidity/comorbidity.jsp). In the present study, all 29 co-morbidities (http://www.hcup-us.ahrq.gov/toolssoftware/comorbidity/Table2-FY12-V3_7.pdf) were included in our analyses. In particular, we examined the likelihood of specific co-morbid conditions among patients with conditions of “insomnia,” “OSA,” and “OSD” and how this likelihood changed over the years.

### Outcome measures

Three outcome measures of hospitalization were considered, MR upon discharge (0, discharged alive; 1, discharged dead), LOS (days) and TC ($). In particular, we were interested in comparing outcomes in patients with and without a particular “sleep disturbance” in 2012 and examining changes over time among patients with a sleep disturbance between the years of 2002 and 2012. For TC trends, values were inflated to 2012 dollars using the consumer price index.

### Covariates

#### Patient-level characteristics

Among patient-level characteristics, we included age (continuous and categorized as 60–64, 65–69, 70–74, 75–79, 80–84, and 85+), sex, race (White, Black, Hispanic, Asian/Pacific Islander, Native American, and Other), median household income for zip code of patient (expressed as quartiles), insurance status (Medicare, Medicaid, Private insurance, self-pay, no charge and other) and admission day (weekday vs. weekend).

#### Hospital-level characteristics

We examined hospital-level characteristics in relation to “sleep disturbance” status and outcomes of healthcare utilization, which included bed size (Small, Medium, Large), ownership of hospital (Government/nonfederal, private non-profit, private investor-owned), location/teaching status of the hospital (rural, urban, non-teaching, urban teaching) and region of the hospital (Northeast, Midwest, South and West).

### Statistical analysis

We used Stata 13.0 (StataCorp, College Station, TX), (STATA, 2013) to analyze data while accounting for survey design complexity based on guidelines outlined by HCUP NIS through incorporation of sampling weights, primary sampling units and strata. Population estimates of proportions, means, and regression coefficients were made (*svy* commands; Stata, 2013). Standard errors were estimated using Taylor series linearization, taking into account sampling weights, strata (combination of five hospital characteristics) and primary sampling units (hospital ID). Multiple regression modeling was also conducted, mainly using linear and logistic regression models, while accounting for sampling design complexity. When waves were combined to examine trends of sleep disturbance rates and outcomes of hospitalization, trend weights were used to ensure redesigned 2012 NIS could be incorporated into the analysis of trends. In order to facilitate analysis of trends using multiple years of NIS data, AHRQ developed new discharge trend weights for the 1993–2011 NIS. These weights were calculated in the same way as the weights for the redesigned 2012 NIS, and were designed for use instead of the original NIS discharge weights for trends analysis. Given that our present trend analysis spans through 2012, starting from 2002, trend weights were used before 2012 data to make estimates comparable to the new 2012 NIS design.

Following our key objectives: **(A)** We first explored proportions of adults 60 years or older who were diagnosed with “insomnia,” “OSA,” or “OSD” as any of 15 possible recorded diagnoses upon discharge. This analysis was conducted from 2002 to 2012, stratifying by sex and age groups. Overall, within each sex and sex-age groups, we assessed trends by conducting logistic regression analyses with year as the only covariate and “sleep disturbance status” status the binary outcome. **(B)** Sleep disturbance status (yes vs. no) among patients for the year 2012—the most recent available year in NIS—were compared by logistic regression with various predictors of sleep disturbance, including patient-level and hospital-level characteristics as well as patient co-morbidities; **(C)** Using 2012 wave of data among older adults aged 60 years or older, we also compared outcomes of hospitalizations for sleep disturbance and non-sleep disturbance patients by logistic and linear regression models with insomnia, OSA, and OSD status as the main predictor of those outcomes, controlling for patient-level, and hospital-level characteristics, and co-morbidities; **(D)** Using data from 2002 through 2012, we conducted a trends analysis of co-morbidities among patients with each of the three classes of sleep disturbance, by estimating proportions with their *SE* and conducting a logistic regression model for each comorbidity with year being the only predictor. Similarly, we examined trends in MR, LOS, and TC using the same methods (i.e., proportion estimation and multiple regression models with year as the only covariates) among patients with each class of sleep disturbance. Another linear model was also conducted to examine the net trend in those three parameters after adjustment for age, sex, and total number of co-morbidity; **(E)** Finally, and using analyses similar to **(C)** but restricting the sample to patients with any sleep disturbance, we ran several regression models testing predictors of hospitalization outcomes in 2012 among older adults, while including insomnia, OSA and OSD status as additional covariates in the model. The secondary analysis of NIS was approved by the institutional review boards of the NIH and Johns Hopkins School of Medicine.

## Results

Of 87,039,711 patients sampled from 2002 to 2012, NIS (weighted mean age ± *SE*: 47.9 ± 0.2, and weighted proportion female ± *SE*: 58.5% ± 0.1, weighted number of discharges: 411,487,801), 35,258,031 were aged 60 years or older (weighted mean age ± SE: 75.37 ± 0.03, and weighted proportion female ± *SE*: 56.0% ± 0.1). The total weighted number of hospital discharges of older adults aged 60 years or older between 2002 and 2012 was estimated at 166,871,086 nationwide. In 2012, the unweighted number of discharges over 60 years of age was 2,825,130; the weighted number was 14,126,650.

Of the 35,258,031 older adults in the unweighted sample (2002–2012), 263,865 had insomnia as “any diagnosis” (weighted percent of discharges ± *SE*: 0.75 ± 0.01); 750,851 had an OSA diagnosis (weighted percent of discharges ± *SE*: 2.14 ± 0.04), 21,814 had a diagnosis of OSD (weighted percent of discharges ± *SE*: 0.06 ± 0.00).

**Figure 1:**
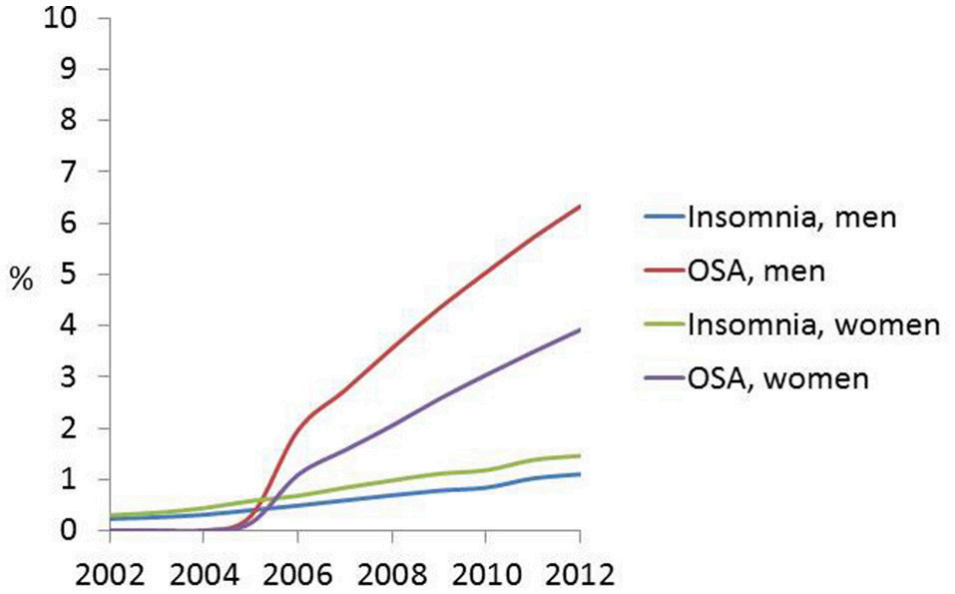
**Sex-specific and overall time trends in insomnia, obstructive sleep apnea**. (OSA) and other sleep disturbances (OSD) (as any diagnosis) rates in the inpatient older adult population (60+y); NIS, 2002–2012.

**Figure 2:**
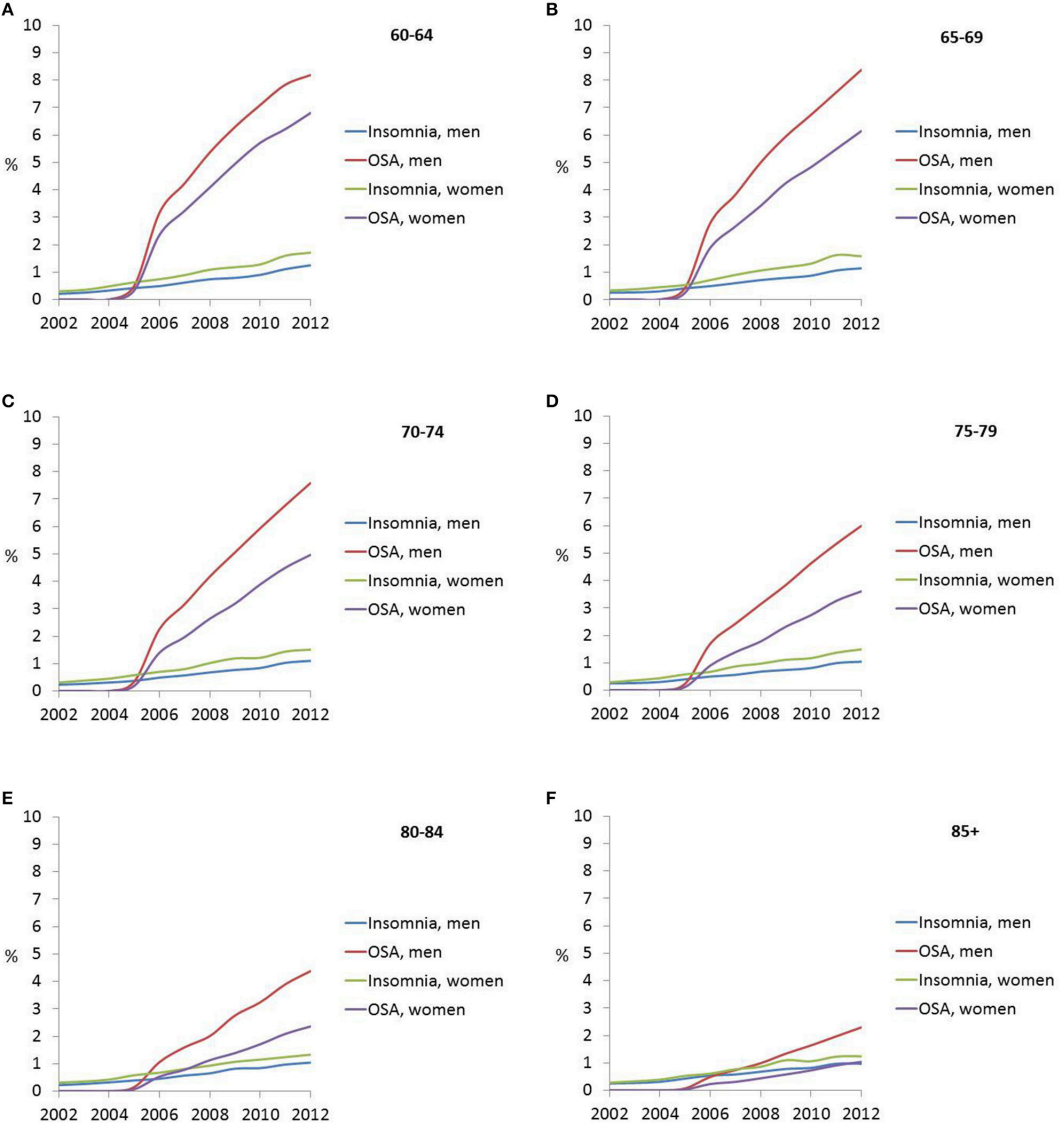
**(A–F)** Sex- and age-specific time trends in insomnia, obstructive sleep apnea. (OSA) and other sleep disturbances (OSD) (as any diagnosis) rates in the inpatient older adult population (60+y); NIS, 2002–2012.

Table S1 and Figure 1 shows the trends in weighted proportions of all three classes of sleep disturbance as any diagnosis, stratifying by sex (**Objective A**). Overall and among men and women, there was an increasing trend in the share of hospitalizations of older adults who were diagnosed with insomnia, OSA, and OSD. A linear trend was particularly observed for insomnia diagnosis, with an estimated rate of 0.27% overall in 2002 rising linearly up to 1.29% in 2012. In contrast, OSA was not diagnosed until 2005 in this inpatient sample (estimated rate of 0.21%) and its rate rose sharply thereafter to reach a level of 5% out of all hospitalizations in 2012. Men tended to have higher rates of OSA compared to women in all years, while the reverse gender difference was true for insomnia (Table S1). Stratifying by both sex and age group, Table S2 and Figures 2A–F shows that in later years (particularly beyond 2009), the young-old (60–64 years) tended to have higher rates of insomnia and OSA compared to older groups and specifically the oldest-old (85+).

Table 1 shows the association of patient and hospital-level characteristics with all three classes of sleep disturbance status among hospitalized older adults (**Objective B**). In general, insomnia status was positively associated with being female, belonging to a younger age group, a White ethnicity, higher income, having Medicare as the primary expected insurance, admission on a weekday, admission to a small bed size (vs. large bed size) or private, investor-owned hospital as opposed to government, and with rural hospitalizations. Geographically speaking, the Northeast had the lowest likelihood of insomnia diagnosis whereas the West had the highest rates. Several co-morbidities were more prevalent in non-insomnia cases compared to insomnia, while others showed no difference. The few exceptions whereby having a co-morbidity was independently linked to higher odds of insomnia included alcohol abuse (OR = 1.17, 95% CI:1.10–1.25, *p* < 0.001), anemia (OR = 1.07, 95% CI:1.04–1.11, *p* < 0.001), depression (OR = 2.73, 95%CI: 2.66–2.81, *p* < 0.001), drug abuse (OR = 1.56, 95%CI:1.44–1.70, *p* < 0.001), hypertension (OR = 1.09, 95%CI:1.06–1.12), hypothyroidism (OR = 1.08, 95%CI: 1.05–1.11, *p* < 0.001), neurological disorders (OR = 1.17, 95%CI:1.13–1.21) and psychoses (OR = 1.98, 95%CI:1.89–2.08, *p* < 0.001).

**Table 1:** Associations of patient and hospital characteristics with insomnia, OSA, and OSD (1, Yes; 0, No) among the US inpatient older adult population: multiple logistic regression models (Unweighted ***N*** = 2,825,283; Weighted ***N*** = 14,126,415)^*^, NIS 2012.

	Unwt. N^*^	Wt. % ±SE^*^	**Insomnia**			**OSA**			**OSD**		
		**60 + y, 2012**	OR^**^	**95% CI**	***P***	OR^**^	**95% CI**	***P***	OR^**^	**95% CI**	***P***
Female	1,677,164	55.1±0.1	1.19	(1.16;1.22)	<0.001	0.53	(0.52;0.54)	<0.001	0.98	(0.90;1.07)	0.71
**AGE GROUP**											
60–64	503,657	16.5±0.1	1			1			1		
65–69	531,122	17.4±0.1	0.91	(0.88;0.95)	<0.001	0.89	(0.87;0.91)	<0.001	0.95	(0.83;1.10)	0.53
70–74	490,322	16.1±0.0	0.90	(0.86;0.94)	<0.001	0.78	(0.77;0.80)	<0.001	1.02	(0.87;1.19)	0.83
74–79	470,935	15.5±0.0	0.89	(0.85;0.93)	<0.001	0.64	(0.62;0.65)	<0.001	0.90	(0.76;1.06)	0.21
80–84	452,445	14.9±0.0	0.84	(0.80;0.88)	<0.001	0.48	(0.46;0.49)	<0.001	0.82	(0.69;0.98)	0.027
85+	597,194	19.6±0.0	0.76	(0.73;0.80)	<0.001	0.26	(0.25;0.27)	<0.001	0.72	(0.61;0.86)	<0.001
**RACE**											
1 = White	2,232,525	77.3±0.4	1			1			1		
2 = Black	312,331	10.8±0.0	0.62	(0.59;0.66)	<0.001	0.88	(0.85;0.92)	<0.001	0.66	(0.56;0.79)	<0.001
3 = Hispanic	194,468	6.7±0.0	0.87	(0.81;0.94)	<0.001	0.63	(0.60;0.67)	<0.001	0.67	(0.53;0.87)	0.002
4 = Asian/Pacific Islander	58,023	2.0±0.0	0.87	(0.78;0.97)	0.014	0.47	(0.43;0.52)	<0.001	0.67	(0.44;1.01)	0.05
5 = Native American	15,399	0.5±0.1	0.84	(0.70;1.00)	0.05	0.93	(0.78;1.11)	0.44	0.31	(0.13;0.75)	0.009
6 = Other	76,738	2.7±0.2	0.96	(0.87;1.06)	0.47	0.72	(0.67;0.79)	<0.001	1.03	(0.80;1.32)	0.81
**MEDIAN HH INCOME FOR ZIP CODE OF PATIENT**											
1st quartile	873,930	29.3±0.5	1			1			1		
2nd quartile	754,442	25.3±0.4	1.01	(0.97;1.05)	0.62	1.09	(1.06;1.12)	<0.001	0.97	(0.85;1.10)	0.65
3rd quartile	710,554	23.8±0.3	1.04	(1.00;1.09)	0.08	1.16	(1.13;1.20)	<0.001	0.95	(0.82;1.09)	0.47
4th quartile	646,730	21.7±0.5	1.08	(1.02;1.14)	0.008	1.20	(1.15;1.24)	<0.001	1.08	(0.93;1.25)	0.33
**INSURANCE STATUS**											
Medicare	2,432,075	80.0±0.2	1			1			1		
Medicaid	100,551	3.3±0.1	0.90	(0.83;0.96)	0.003	0.69	(0.66;0.73)	<0.001	0.68	(0.52;0.91)	0.008
Private insurance	409,617	13.4±0.2	0.97	(0.93;1.02)	0.25	1.01	(0.98;1.03)	0.49	1.10	(0.96;1.26)	0.16
Self-pay	39,753	1.3±0.0	0.71	(0.61;0.82)	<0.001	0.49	(0.45;0.54)	<0.001	0.91	(0.60;1.39)	0.67
No charge	3441	0.1±0.0	1.03	(0.73;1.46)	0.87	0.56	(0.41;0.76)	<0.001	1.08	(0.34;3.41)	0.89
Other	54,012	1.8±0.1	0.86	(0.78;0.95)	0.003	0.86	(0.81;0.90)	<0.001	0.84	(0.60;1.17)	0.29
**ADMISSION DAY**											
Weekday	2,440,195	80.1±0.0	1			1			1		
Weekend	605,477	19.9±0.1	0.95	(0.93;0.98)	0.001	0.94	(0.92;0.95)	<0.001	0.91	(0.81;1.01)	0.08
**CO-MORBIDITY**											
AIDS	2002	0.1±0.0	0.61	(0.34;1.10)	0.10	0.47	(0.34;0.65)	<0.001	0.70	(0.10;5.02)	0.72
Alcohol Abuse	80,337	2.6±0.0	1.17	(1.10;1.25)	<0.001	0.59	(0.56;0.61)	<0.001	0.72	(0.55;0.95)	0.018
Deficiency Anemia	670,473	22.0±0.1	1.07	(1.04;1.11)	<0.001	0.92	(0.91;0.94)	<0.001	0.97	(0.86;1.09)	0.57
Rheumatoid arthritis/collagen vascular disease	108,420	3.6±0.0	1.01	(0.95;1.06)	0.82	1.18	(1.15;1.22)	<0.001	1.14	(0.93;1.39)	0.20
Chronic blood loss anemia	39,495	1.3±0.0	0.98	(0.87;1.10)	0.71	0.79	(0.74;0.84)	<0.001	1.01	(0.68;1.50)	0.96
Congestive heart failure	420,652	13.8±0.1	0.77	(0.74;0.80)	<0.001	1.37	(1.34;1.39)	<0.001	0.80	(0.69;0.92)	0.002
Chronic pulmonary disease	720,894	23.7±0.1	0.97	(0.94;0.99)	<0.001	1.79	(1.76;1.81)	<0.001	1.02	(0.93;1.13)	0.63
Coagulopathy	176,187	5.8±0.0	0.80	(0.76;0.85)	<0.001	0.88	(0.86;0.91)	<0.001	0.61	(0.47;0.78)	<0.001
Depression	356,534	11.7±0.1	2.73	(2.66;2.81)	<0.001	1.37	(1.35;1.43)	<0.001	1.99	(1.80;2.21)	<0.001
Diabetes, uncomplicated	802,141	26.3±0.1	0.73	(0.71;0.76)	<0.001	1.50	(1.48;1.52)	<0.001	0.84	(0.76;0.93)	0.001
Diabetes, complicated	179,761	5.9±0.1	0.69	(0.65;0.73)	<0.001	1.39	(1.35;1.43)	<0.001	0.79	(0.64;0.98)	0.036
Drug abuse	29,617	0.1±0.0	1.56	(1.44;1.70)	<0.001	0.72	(0.68;0.77)	<0.001	8.52	(7.02;10.34)	<0.001
Hypertension	2,109,734	69.3±0.1	1.09	(1.06;1.12)	<0.001	1.21	(1.20;1.23)	<0.001	1.02	(0.93;1.12)	0.62
Hypothyroidism	512,730	16.8±0.1	1.08	(1.05;1.11)	<0.001	1.22	(1.20;1.24)	<0.001	1.15	(1.03;1.28)	0.013
Liver disease	72,826	2.4±0.0	0.91	(0.84;0.97)	0.009	0.86	(0.83;0.90)	<0.001	0.86	(0.63;1.17)	0.34
Lymphoma	34,835	1.1±0.0	0.94	(0.84;1.04)	0.23	0.84	(0.79;0.90)	<0.001	0.81	(0.51;1.28)	0.38
Fluid/electrolyte disorders	897,369	29.5±0.1	0.92	(0.90;0.95)	<0.001	0.82	(081;0.83)	<0.001	0.83	(0.75;0.92)	0.001
Metastatic cancer	90,230	3.0±0.0	0.83	(0.77;0.89)	<0.001	0.52	(0.50;0.55)	<0.001	0.61	(0.44;0.84)	0.003
Neurological disorders	301,899	9.9±0.0	1.17	(1.13;1.21)	<0.001	1.13	(1.10;1.15)	<0.001	1.52	(1.35;1.71)	<0.001
Obesity	329,904	10.8±0.1	0.90	(0.86;0.93)	<0.001	5.15	(5.05;5.25)	<0.001	1.36	(1.20;1.54)	<0.001
Paralysis	88,068	2.9±0.0	0.93	(0.86;1.00)	0.042	0.68	(0.65;0.71)	<0.001	1.18	(0.91;1.54)	0.20
Peripheral vascular disorders	292,442	9.6±0.0	0.80	(0.76;0.83)	<0.001	0.83	(0.81;0.85)	<0.001	0.85	(0.72;1.01)	0.07
Psychoses	111,610	3.7±0.0	1.98	(1.89;2.08)	<0.001	0.95	(0.91;0.98)	0.003	1.75	(1.47;2.10)	<0.001
Pulmonary circulation disorders	97,609	3.2±0.0	0.79	(0.76;0.83)	<0.001	1.70	(1.65;1.75)	<0.001	0.98	(0.75;1.28)	0.90
Renal failure	562,107	18.5±0.1	0.73	(0.71;0.76)	<0.001	1.14	(1.12;1.17)	<0.001	0.70	(0.61;0.80)	<0.001
Non-metastatic cancer	91,610	3.0±0.0	0.97	(0.91;1.03)	0.28	0.72	(0.70;0.75)	<0.001	0.65	(0.47;0.92)	0.014
Peptic ulcer	1222	0.0±0.0	1.09	(0.67;1.78)	0.72	0.68	(0.48;0.95)	0.025	4.58	(1.72;12.20)	0.002
Valvular disease	176,782	5.8±0.1	0.98	(0.93;1.04)	0.53	0.99	(0.96;1.02)	0.40	1.14	(0.94;1.39)	0.18
Weight loss	193,961	6.4±0.1	0.84	(0.80;0.89)	<0.001	0.45	(0.44;0.47)	<0.001	0.90	(0.74;1.09)	0.29
**BED SIZE**											
Small	458,999	15.0±0.0	1			1			1		
Medium	801,171	26.3±0.0	0.95	(0.89;1.02)	0.17	1.06	(1.00;1.12)	0.048	0.96	(0.80;1.16)	0.70
Large	1,785,505	58.6±0.0	0.92	(0.87;0.98)	0.010	1.11	(1.05;1.17)	<0.001	0.80	(0.67;0.95)	0.009
**OWNERSHIP OF HOSPITAL**											
Government, nonfederal	321,524	10.6±0.0	1			1			1		
Private, non-profit	2,275,706	74.7±0.0	1.02	(0.94;1.10)	0.71	1.12	(1.04;1.20)	0.003	1.26	(1.04;1.53)	0.019
Private, investor-own	448,445	14.7±0.0	1.11	(1.01;1.22)	0.033	0.98	(0.90;1.07)	0.61	1.10	(0.86;1.40)	0.47
**LOCATION/TEACHING STATUS**											
Rural	404,639	13.3±0.0	1			1			1		
Urban, non-teaching	1,232,393	40.5±0.0	0.80	(0.74;0.85)	<0.001	1.23	(1.16;1.32)	<0.001	0.77	(0.65;0.91)	0.002
Urban, teaching	1,408,643	46.3±0.0	0.82	(0.76;0.88)	<0.001	1.46	(1.37;1.56)	<0.001	0.70	(0.59;0.83)	<0.001
**REGION OF HOSPITAL**											
Northeast	612,680	20.1±0.4	1			1			1		
Midwest	715,874	23.5±0.4	1.27	(1.17;1.38)	<0.001	1.36	(1.27;1.45)	<0.001	2.00	(1.67;2.40)	<0.001
South	1,175,080	38.6±0.5	1.44	(1.33;1.56)	<0.001	1.18	(1.11;1.25)	<0.001	1.75	(1.47;2.07)	<0.001
West	542,041	17.8±0.3	1.61	(1.48;1.76)	<0.001	1.12	(1.04;1.21)	<0.001	1.58	(1.29;1.93)	<0.001
Female	1,677,164	55.1±0.1	1.19	(1.16;1.22)	<0.001	0.53	(0.52;0.54)	<0.001	0.98	(0.90;1.07)	0.71

NIS, Nationwide Inpatient Sample; OSA, Obstructive Sleep Apnea; OSD, Other Sleep Disturbances; Unwt, Unweighted; Wt, Weighted.

* Sample sizes and % were based on data availability per covariate among older adults 60 + y in the NIS 2012. Unweighted and weighted N are based on simultaneous availability of data in the logistic regression models.

** Odds ratios (OR) are estimated from a multiple logistic regression model with their 95% confidence interval (CI) and thus are multivariate-adjusted for all covariates included in the model. OR are interpreted as the odds of having the outcome of interest among the exposed group(s) relative to the odds of having the outcome of interest among the unexposed group (referent category), controlling for all other covariates in the model.

In contrast, the odds of an OSA diagnoses was greater among men, but lower with age, significantly higher among Whites and patients with higher income, and generally higher among Medicare beneficiaries. The odds for diagnosis with OSA was lower in weekend admissions as was insomnia, but the odds was higher with bed size, highest in private non-profit and urban hospitals, while remaining lowest in the Northeast. Several co-morbidities had independent positive associations with OSA and many of those intersected with those observed with insomnia. These included rheumatoid arthritis, congestive heart failure, depression, diabetes (both uncomplicated and complicated), hypertension, hypothyroidism, neurological disorders, pulmonary circulation disorders, renal failure, and most importantly obesity (OR = 5.15, 95%CI: 5.05–5.25, *p* < 0.001).

The likelihood of OSD diagnoses was not modified by gender or income though was still lowest among the oldest-old, and highest among Whites. Hospital-level characteristics were associated with OSD in a way similar to that of OSA, with the odds doubled in the Midwest compared to the Northeast. Most notably, while alcohol abuse was inversely related to OSD, drug abuse was among the strongest predictors of OSD (OR = 8.52, 95%CI:7.02–10.34, *p* < 0.001) followed by peptic ulcer (OR = 4.58, 95%CI:1.72–12.20, *p* = 0.002). Other co-morbidities that were positively linked to OSD included depression, hypertension, neurological disorders, obesity, and psychoses.

Table 2 displays findings from multiple regression models testing associations between the three classes of sleep disturbance as predictors of MR, LOS, and TC, while adjusting for individual-level and hospital-level characteristics (**Objective C**). Generally, diagnosis with any of the three sleep disturbances was associated with lower MR and lower TC compared to non-diagnosis. LOS was higher for insomnia vs. non-insomnia and for OSD vs. non-OSD, though the reverse pattern was observed for OSA status.

**Table 2:** **Outcomes of healthcare utilization (MR, LOS, and TC) among older adults by sleep disturbance status, patient-level, and hospital-level characteristics: multiple logistic and OLS regression models, NIS 2012**.

	**Model 1: MR**			**Model 2: LOS (days)**			**Model 3: TC ($)**		
	OR^**^	**95% CI**	***P***	β^**^	**(SE)**	***P***	β^**^	**(SE)**	***P***
**INSOMNIA STATUS**									
Non-insomnia	1			–			–		
Insomnia (any diagnosis)	0.36	(0.32;0.40)	<0.001	+0.44	(0.05)	<0.001	–4715	(339)	<0.001
**OSA STATUS**									
Non-OSA	1			–			–		
OSA (any diagnosis)	0.58	(0.56;0.61)	<0.001	–0.34	(0.02)	<0.001	–2125	(316)	<0.001
**OSD STATUS**									
Non-OSD	1			–			–		
OSD (any diagnosis)	0.35	(0.23;0.54)	<0.001	+0.40	(0.12)	0.001	–5853	(787)	<0.001
Unwt. N^*^		2,824,671			2,825,124		2,767,001		
Wt. N^*^		14,123,355			14,125,620		13,835,005		

LOS, Length of stay; MR, mortality Risk; NIS, Nationwide Inpatient Sample; OSA, Obstructive Sleep Apnea; OSD, Other Sleep Disturbances; TC, Total Charges; Unwt, Unweighted; Wt, Weighted.

* Weighted and unweighted sample sizes are based on multiple logistic regression models with outcome being “all sleep disturbances.”

** Odds ratios (OR) are estimated from a multiple logistic regression model with their 95% confidence interval (CI) and linear regression coefficients (β) are estimated from multiple ordinary least square (OLS) models with their standard errors (SE) and thus are multivariate-adjusted for all covariates included in the model. OR are interpreted as the odds of mortality among the exposed group(s) relative to the odds of mortality among the unexposed group (referent category), controlling for all other covariates in the model. β is the estimated adjusted difference in LOS or TC between referent and exposure category(ies). All models controlled for sex, age, race, income, insurance status, admission day, co-morbidity, hospital bed size, ownership of hospital, location/teaching status, and region of the hospital.

Among hospitalized older adults who were diagnosed with each of the three classes of sleep disturbances, trends in co-morbidities from 2002 to 2012 are presented in Table S3 (**Objective D**). Overall, the average number of co-morbidities increased steadily from 2.28 in 2002 to 3.10 in 2012 for insomnia, from 3.11(2005) to 3.87(2012) for OSA and 1.92(2002) to 3.19(2012) for OSD. Some of the most prevalent co-morbidities (>10% in 2012) in insomnia patients included deficiency anemia (~14% in 2002 → 21% in 2012), chronic pulmonary disorder (22% in 2002 → 24% in 2012), depression (20% in 2002 → 27% in 2012), uncomplicated diabetes (~15% in 2002 → 21% in 2012), hypertension (52% in 2002 → 70% in 2012), hypothyroidism (14% in 2002 → 20% in 2012), fluid/electrolyte disorder (18% in 2002 → 27% in 2012), neurological disorders (6.3% in 2002 → 12.5% in 2012), and renal failure (3% in 2002 → 12% in 2012). Except for congestive heart failure, the rates among insomnia patients rose over time for most co-morbidities. Among OSA patients, the most prevalent co-morbidities included those observed among insomnia patients in addition to obesity (42% in 2012) and congestive heart failure (19% in 2012). Despite similar comorbidity patterns between the three classes of sleep disturbance, OSD had the highest co-morbidity with drug abuse compared to insomnia and OSA (7.5% in 2012 vs. 1.8 for insomnia and 0.9% in OSA).

In parallel with the rise in co-morbidities among older adult patients with sleep disturbance, TC rose steadily from an average of $22,250/admission in 2002 to $38,177/admission in 2012 for insomnia with similar mean annual changes observed for OSA and OSD ($1414/year for OSA, $1635/year for OSD and $1722/year for insomnia). The rate of increase in cost was slightly attenuated but remained significant after adjustment for age, sex, and total number of co-morbidities. However, both MR and LOS have been markedly reduced over-time, with the exception of MR for OSD (*p* > 0.05). In particular, MR dropped steadily from 1.5% in 2002 to 1.0% in 2012 among insomnia patients while mean LOS was reduced from 6.1 days in 2002 to 5.4 days in 2012. Those rates of decrease over time were even more marked after control was made on age, sex, and total number of co-morbidities (Table S4, **Objective D**).

Using data from 2012 on inpatient older adults with “any sleep disturbance,” we tested predictors of outcomes of hospitalization (Table 3, **Objective E**). MR was lower among women who also had a significantly lower TC. MR increased linearly with age, with longer LOS but lower TC observed in the older groups. TC was also higher among Hispanics and other ethnic groups compared to Whites, with Blacks having significantly lower MR and higher LOS compared to Whites. The 4th quartile of income was significantly more expensive in terms of TC compared to the 1st quartile, though LOS was shorter and MR lower.

**Table 3:** **Outcomes of healthcare utilization (MR, LOS, and TC) among older adults with “any sleep disturbance,” patient-level and hospital-level characteristics: multiple logistic and OLS regression models, NIS 2012**.

			**Model 1: MR**			**Model 2: LOS(days)**			**Model 3: TC ($)**		
	Unwt. N^*^	Wt. % ±SE^*^	**OR**	**95% CI**	***P***	**β**	**(SE)**	***P***	**β**	**(SE)**	***P***
Insomnia (yes vs. no)	39,430	20.5±0.3	0.35	(0.18;0.69)	0.003	+0.09	(0.10)	0.34	−3424	(1143)	0.003
OSA (yes vs. no)	152,299	79.2±0.3	0.56	(0.28;1.12)	0.10	−0.62	(0.11)	< 0.001	+2222	(1126)	0.049
Other disturbance (yes vs. no)	2249	1.3±0.0	0.35	(0.15;0.82)	0.015	+0.07	(0.64)	0.64	−4788	(1259)	< 0.001
Female	90,673	47.2±0.2	0.91	(0.84;1.00)	0.045	−0.04	(0.03)	0.18	−2984	(292)	< 0.001
**AGE**											
60–64	45,224	23.5±0.1	1			–			–		
65–69	45,694	23.8±0.1	1.29	(1.12;1.51)	0.001	+0.03	(0.04)	0.44	+507	(447)	0.26
70–74	37,005	19.2±0.1	1.64	(1.40;1.93)	< 0.001	+0.10	(0.05)	0.046	−1106	(527)	0.036
74–79	28,279	14.7±0.1	1.82	(1.54;2.14)	< 0.001	+0.18	(0.05)	0.001	−2752	(552)	< 0.001
80–84	20,166	10.5±0.1	2.22	(1.87;2.62)	< 0.001	+0.33	(0.06)	< 0.001	−4413	(607)	< 0.001
85+	15,956	8.3±0.1	3.07	(2.58;3.65)	< 0.001	+0.21	(0.08)	0.011	−9022	(653)	< 0.001
**RACE**											
1 = White	147,852	81.6±0.4	1			–			–		
2 = Black	18,194	10.0±0.3	0.82	(0.71;0.95)	0.007	+0.15	(0.06)	0.011	+442	(946)	0.64
3 = Hispanic	8542	4.7±0.2	1.00	(0.83;1.20)	0.97	+0.03	(0.09)	0.70	+6356	(1449)	< 0.001
4 = Asian/Pacific Islander	1776	1.0±0.1	1.04	(0.73;1.49)	0.81	+0.29	(0.17)	0.09	+3430	(2743)	0.21
5 = Native American	961	0.5±0.1	1.09	(0.66;1.80)	0.73	−0.01	(0.22)	0.97	−7306	(3649)	0.045
6 = Other	3792	2.1±0.2	0.91	(0.68;1.20)	0.49	+0.23	(0.11)	0.039	+5045	(2487)	0.043
**MEDIAN HH INCOME FOR ZIP CODE OF PATIENT**											
1st quartile	51,128	27.1±0.5	1			–			–		
2nd quartile	48,773	25.8±0.4	0.95	(0.86;1.06)	0.37	−0.15	(0.04)	< 0.001	−181	(553)	0.74
3rd quartile	48,015	25.4±0.4	0.85	(0.76;0.95)	0.005	−0.19	(0.05)	< 0.001	+1045	(657)	0.11
4th quartile	41,027	21.7±0.6	0.80	(0.70;0.90)	< 0.001	−0.29	(0.05)	< 0.001	+4623	(1001)	< 0.001
**INSURANCE STATUS**											
Medicare	147,649	76.9±0.2	1			–			–		
Medicaid	5650	2.9±0.7	1.34	(1.04;1.74)	0.026	+0.83	(0.18)	< 0.001	−1050	(956)	0.27
Private insurance	33,410	17.4±0.2	1.17	(1.00;1.35)	0.046	−0.05	(0.04)	0.18	+1622	(556)	0.004
Self-pay	1543	0.8±0.0	1.18	(0.66;1.96)	0.64	+1.06	(0.28)	< 0.001	+3232	(3804)	0.40
No charge	166	0.1±0.1	0.69	(0.09;5.13)	0.72	+1.69	(1.05)	0.11	−6771	(3856)	0.08
Other	3618	1.9±0.1	2.30	(1.77;2.99)	< 0.001	+0.15	(0.11)	0.18	+1076	(1159)	0.35
**ADMISSION DAY**											
Weekday	157,416	81.8±0.1	1			–			–		−
Weekend	34,908	18.2±0.1	1.41	(1.29;1.54)	< 0.001	−0.12	(0.03)	0.001	−6191	(359)	< 0.001
**CO-MORBIDITY**											
AIDS	64	0.0±0.0	–	–		−0.13	(0.70)	0.86	+5272	(10,096)	0.60
Alcohol Abuse	4114	2.1±0.0	1.06	(0.80;1.41)	0.66	+0.37	(0.10)	< 0.001	−2616	(1,011)	0.010
Deficiency Anemia	40,039	20.8±0.2	0.94	(0.85;1.03)	0.21	+0.96	(0.04)	< 0.001	+5761	(449)	< 0.001
Rheumatoid arthritis/collagen vascular disease	7661	4.0±0.1	1.02	(0.83;1.26)	0.85	+0.02	(0.06)	0.70	−946	(606)	0.12
Chronic blood loss anemia	2023	1.1±0.0	0.91	(0.64;1.30)	0.62	+1.15	(0.12)	< 0.001	+10,899	(1419)	< 0.001
Congestive heart failure	33,552	17.4±0.1	1.61	(1.47;1.77)	< 0.001	+0.68	(0.04)	< 0.001	−1934	(402)	< 0.001
Chronic pulmonary disease	66,136	34.3±0.2	1.24	(1.14;1.34)	< 0.001	+0.34	(0.03)	< 0.001	+2705	(349)	< 0.001
Coagulopathy	9448	4.9±0.1	1.77	(1.55;2.02)	< 0.001	+1.24	(0.07)	< 0.001	+20,020	(1666)	< 0.001
Depression	35,872	18.7±0.2	0.82	(0.73;0.92)	0.001	−0.06	(0.03)	0.049	−1862	(354)	< 0.001
Diabetes, uncomplicated	68,935	35.8±0.2	1.07	(0.98;1.17)	0.11	+0.07	(0.03)	0.009	−1274	(303)	< 0.001
Diabetes, complicated	16,689	8.7±0.1	0.81	(0.71;0.95)	0.007	+0.55	(0.05)	< 0.001	+118	(657)	0.86
Drug abuse	2175	1.1±0.0	0.71	(0.43;1.15)	0.16	+0.16	(0.12)	0.16	−1799	(1265)	0.16
Hypertension	143,908	74.8±0.1	0.78	(0.72;0.95)	< 0.001	−0.08	(0.03)	0.005	+2170	(337)	< 0.001
Hypothyroidism	35,425	18.4±0.1	0.86	(0.77;0.95)	0.005	+0.02	(0.03)	0.50	+78	(324)	0.81
Liver disease	4538	2.4±0.0	1.15	(0.90;1.46)	0.26	−0.06	(0.08)	0.47	−4404	(930)	< 0.001
Lymphoma	4528	0.9±0.0	1.89	(1.42;2.52)	< 0.001	+0.72	(0.16)	< 0.001	+259	(1606)	0.87
Fluid/electrolyte disorders	48,524	25.2±0.2	2.29	(2.10;2.50)	< 0.001	+1.26	(0.04)	< 0.001	+8318	(537)	< 0.001
Metastatic cancer	3131	1.6±0.0	3.03	(2.48;2.50)	< 0.001	+0.91	(0.12)	< 0.001	+3130	(1265)	0.013
Neurological disorders	19,151	10.0±0.1	1.24	(1.10;1.40)	0.001	+0.38	(0.05)	< 0.001	−539	(537)	0.32
Obesity	67,602	35.2±0.2	1.00	(0.91;1.09)	0.97	+0.48	(0.03)	< 0.001	+4033	(357)	< 0.001
Paralysis	4230	2.2±0.0	1.31	(1.03;1.67)	0.025	+3.56	(0.17)	< 0.001	+11,422	(1620)	< 0.001
Peripheral vascular disorders	17,437	9.1±0.1	1.06	(0.94;1.21)	0.33	+0.06	(0.04)	0.12	+3507	(499)	< 0.001
Psychoses	8623	4.5±0.1	0.86	(0.70;1.07)	0.18	+0.58	(0.08)	< 0.001	−1471	(716)	0.040
Pulmonary circulation disorders	10,263	5.3±0.1	1.85	(1.63;2.09)	< 0.001	+0.88	(0.06)	< 0.001	+4340	(776)	< 0.001
Renal failure	40,066	20.8±0.2	1.58	(1.44;1.73)	< 0.001	+0.13	(0.04)	< 0.001	−1839	(423)	< 0.001
Non-metastatic cancer	4289	2.2±0.0	1.79	(1.46;2.18)	< 0.001	+0.40	(0.09)	< 0.001	−2250	(841)	0.007
Peptic ulcer	60	0.0±0.0	–	−		−0.10	(0.58)	0.86	−1823	(5881)	0.22
Valvular disease	10,923	5.7±0.1	1.01	(0.87;1.16)	0.93	+0.06	(0.05)	0.26	−685	(556)	0.22
Weight loss	6089	3.2±0.1	2.52	(2.18;2.92)	< 0.001	+3.00	(0.13)	< 0.001	+22,037	(2211)	< 0.001
**BED SIZE**											
Small	26,976	14.0±0.4	1			–			–		
Medium	49,997	26.0±0.6	1.22	(1.05;1.41)	0.009	+0.08	(0.08)	0.30	+3889	(1263)	0.002
Large	115,351	60.0±0.7	1.36	(1.19;1.56)	< 0.001	+0.52	(0.07)	< 0.001	+11,453	(1278)	< 0.001
**OWNERSHIP OF HOSPITAL**											
Government, nonfederal	17,874	9.3±0.4	1			–			–		
Private, non-profit	149,417	77.7±0.6	0.97	(0.83;1.12)	0.83	−0.29	(0.08)	< 0.001	799	(1798)	0.66
Private, investor-own	25,033	13.0±0.4	0.88	(0.73;1.06)	0.18	−0.12	(0.09)	0.18	19,837	(1939)	< 0.001
**LOCATION/TEACHING STATUS**											
Rural	21,853	11.4±0.4	1			–			–		
Urban, non-teaching	74,897	38.9±0.7	0.98	(0.85;1.14)	0.83	+0.51	(0.06)	< 0.001	12,927	(1003)	< 0.001
Urban, teaching	95,574	49.7±0.7	1.02	(0.89;1.18)	0.73	+0.90	(0.07)	< 0.001	21,308	(1343)	< 0.001
**REGION OF HOSPITAL**											
Northeast	31,994	16.6±0.5	1			–			–		
Midwest	54,484	28.3±0.7	0.98	(0.86;1.13)	0.82	−0.51	(0.07)	< 0.001	−6613	(1789)	< 0.001
South	72,882	37.9±0.7	1.02	(0.90;1.16)	0.76	−0.46	(0.07)	< 0.001	−5905	(1818)	0.001
West	32,964	17.1±0.5	1.09	(0.94;1.26)	0.26	−0.79	(0.09)	< 0.001	+10,847	(2562)	< 0.001
Unweighted sample	192,324			177,508			177,632			173,859	
Weighted sample	961,620			887,540			888,160			869,295	

AIDS, Acquired Immune deficiency Syndrome; CHF, Congestive Heart Failure; LOS, Length of stay; MR, mortality Risk; NIS, Nationwide Inpatient Sample; OSA, Obstructive Sleep Apnea; OSD, Other Sleep Disturbances; TC, Total Charges; Unwt, Unweighted; Wt, Weighted.

* Sample of older adults with any sleep disturbance in 2012 having complete data on each covariates entered in the model.

## Discussion

This study is the first to utilize a large national healthcare database to comprehensively examine trends in sleep disturbance diagnoses of insomnia, OSA, and OSD among hospitalized older adults in the United States and whether co-morbidities and outcomes are related to disturbance status. Moreover, this study tested both patient-level and hospital-level predictors of sleep disturbance status and outcomes of hospitalization.

Our study observed that the proportion of older adults with a sleep diagnosis has increased significantly over the last decade. Insomnia and OSA diagnoses in older adults have more than tripled from 2002 to 2012, while diagnoses for OSD have almost doubled over this period. A study using the National Health Interview Survey (Ford et al., 2015) observed similar increasing rate trends specifically for insomnia from 2002 (17.5%) and 2012 (19.2%) with adults 18 years and older. The study also observed greater rate changes over time for the youngest-old (55–64 years: 22.1–24.2%) and middle-old (65–74 years: 18.6–21.3%) than the oldest-old (75 years and older: 20.5–20.7%) age groups. The current study expands upon these findings, in that we observed that the youngest-old (60–64 years) tended to have greater rates of change for both insomnia and OSA than the older age groups (65 years and older). A possible explanation for this finding is that a percentage of the oldest-old with sleep disturbances and comorbid health conditions may have died at earlier ages. Thus, a survivor effect may be occurring where the oldest-old adults with sleep disturbances are a selected group of individuals with less severe sleep disturbances and/or comorbid medical conditions. Nevertheless, a growing body of evidence has shown that (short or long) sleep duration (Cohen-Mansfield and Perach, 2012; Ensrud et al., 2012; Garde et al., 2013; Jung et al., 2013; Kakizaki et al., 2013; Kurina et al., 2013; Yeo et al., 2013; Azevedo Da Silva et al., 2014; Benito-León et al., 2014; Duggan et al., 2014; Pan et al., 2014; Rod et al., 2014; Xiao et al., 2014; Cai et al., 2015; Hall et al., 2015), sleep disturbances (Ensrud et al., 2012; Omachi et al., 2012; Rod et al., 2014), and obstructive sleep apnea (OSA; Ge et al., 2013; Lee et al., 2013; Lockhart et al., 2013; Muraja-Murro et al., 2013; Stanchina et al., 2013; Kendzerska et al., 2014; Louis et al., 2014; Marshall et al., 2014) may predict all-cause (Seicean et al., 2011; Ensrud et al., 2012; Howrey et al., 2012; Johansson et al., 2012; Nieto et al., 2012; Omachi et al., 2012; Garde et al., 2013; Ge et al., 2013; Jung et al., 2013; Kakizaki et al., 2013; Kurina et al., 2013; Lee et al., 2013; Lockhart et al., 2013; Muraja-Murro et al., 2013; Yeo et al., 2013; Louis et al., 2014; Marshall et al., 2014; Pan et al., 2014; Rod et al., 2014; Xiao et al., 2014; Hall et al., 2015; Rahman and Adjeroh, 2015), cardiovascular- (Nieto et al., 2012; Garde et al., 2013; Ge et al., 2013; Kakizaki et al., 2013; Yeo et al., 2013; Azevedo Da Silva et al., 2014; Marshall et al., 2014; Rod et al., 2014; Xiao et al., 2014), cancer- (Yeo et al., 2013; Marshall et al., 2014; Rod et al., 2014; Xiao et al., 2014; Rahman and Adjeroh, 2015), and dementia-specific (Cai et al., 2015) mortality. Another explanation is the increased number of co-morbidities with age that are deemed more serious to report than sleep disturbances thus those diagnoses are often missed at older ages given that only 15 diagnoses are allowed for this analysis. Generally speaking, several reasons have been proposed for the increasing sleep disturbances rates including, but not limited to, the following: (1) increased number of health conditions; (2) increase in obesity rates; (3) increase in perceived stress; (4) poor sleep habits/hygiene; (5) increase in shift work; (6) increased use of electronic devices, particularly close to bedtime, a behavior that has become salient in the US, though possibly less so among older adults (Pallesen et al., 2014; Ford et al., 2015).

While in-hospital LOS and MR among admissions with sleep disturbances has decreased, TC has increased over time. Specifically, insomnia-related hospital charges have increased from $22,250 to $38,177, OSA-related hospital charges have increased from $37,561 to $46,518, and OSD-related hospital charges have increased from $18,264 to $35,450, with those trends only partly explained by age, sex and total comorbidity distribution changes over the years. In comparison to younger adults, older adults have shown to incur direct (inpatient, outpatient, pharmacy, and emergency room) charges greater than $1253 for untreated sleep disturbances, such as insomnia (Ozminkowski et al., 2007). Particularly in light of recent data suggesting poorer health outcomes and increased rates of readmission in those individuals suffering with untreated sleep disorders, increased efforts aimed at training providers delivering both the acute (inpatient) and primary/preventive health care may assist in reducing hospitalization costs. The reduction in MR and LOS may be explained by the improved in-hospital procedures to stabilize/improve health co-morbidities (e.g., hypertension and diabetes) often associated with sleep disturbances. One study partially supports this explanation, by observing that in-hospital mortality risk appeared to decline significantly in OSA patients after adjusting for health comorbidities and demographic characteristics (Lyons et al., 2015). Consequently, it is possible that increased costs may be a result of implementing a variety of in-hospital procedures for stabilizing/improving older adults' health status, especially if they have complex etiologies, including sleep disturbances and other co-morbidities, that leads to a complex case-mix requiring multifaceted interventions. The increased costs, but reduced MR and LOS, may also reflect enhanced techniques for early disease detection and copious treatment resources.

Our study observed several patient characteristic differences across the three classes of sleep disturbances. Over the last decade men tended to have higher rates for OSA, while women tend to have higher rates for insomnia. These gender differences in OSA and insomnia rates have been supported in previous studies (Ohayon et al., 2004; Wolkove et al., 2007; Salas et al., 2014). In terms of hospitalization outcomes, women with any type of sleep disturbance tended to have lower MR and lower TC than men. It is unclear what physiological, sociological, and/or psychological factors explain the gender differences in OSA, which could assist in developing an approach for improving hospitalization outcomes associated with OSA in men. However, it has been proposed that estrogen deficiency, particularly during the peri-menopausal period, may account for the higher number of women with insomnia (Ohayon et al., 2004; Wolkove et al., 2007).

Interestingly, we observed that older whites had consistently higher rates of insomnia, OSA, and OSD over the last decade than older Blacks and older Hispanics. Furthermore, older Blacks with any type of sleep disturbance appeared to have lower MR, but higher LOS compared to older Whites. Previous literature has observed mixed findings regarding the racial differences in sleep disturbance rates. While some studies have supported our findings that sleep disturbances appear to be higher in older Whites than older Blacks (Redline et al., 1997; Durrence and Lichstein, 2006), other studies have observed that sleep disturbances appear to be higher in older Blacks than older Whites (Foley et al., 1999; Ancoli-Israel et al., 2002). A potential explanation for this inconsistent finding is the different methodological approaches. The current study uses clinical diagnostic codes, such as ICD-9-CM, given by a health provider to estimate sleep disturbance; however, it is unclear what diagnostic tests were given to assess the symptoms/signs common to each sleep disturbance. Many of the previous studies have estimated sleep disturbance, particularly OSA, after participants have completed clinical evaluation, standardized questionnaires, an in-lab overnight sleep study, and/or in-home sleep testing (HST; Salas et al., 2014). Furthermore, many of the previous studies have actively recruited participants within the community, while this study is accounting for individuals in the community that sought medical care. Given Blacks are less likely than Whites to utilize health care services and/or more likely to utilize health care resources with severe symptomology (Weech-Maldonado et al., 2014), our study may be underestimating the rates of sleep disturbances and the MR associated with sleep disturbances within older Blacks. However, our finding may further provides support that sleep disturbances, particularly in Blacks, may be underdiagnosed in the health care setting (Kapur et al., 2002; Benca, 2014; Salas et al., 2014). This is corroborated with findings on income differentials in the rates of sleep disturbance, whereby for both insomnia and OSA, higher income individuals had a higher rate, suggesting higher access to health care, particularly for OSA which requires a sleep study as opposed to insomnia which is only assessed through a clinical diagnosis and patient history.

Primary insurance also appeared to be associated with sleep disturbances. Older adults with Medicare as their primary insurance had higher rates across the classes of sleep disturbances than older adults with a different primary insurance. However, Medicare beneficiaries with any type of sleep disturbance had lower MR, lower LOS, and lower TC in comparison with some of the other primary insurance programs (i.e., Medicaid, private, and self-pay). Since there are several programs available under Medicare (e.g., Medicare Advantage program (Weech-Maldonado et al., 2014), future studies should explore differences in sleep disturbance rates and hospitalization outcomes across the Medicare plans.

Similar to previous literature, the current study found that sleep disturbances are associated with cardiovascular risk factors (e.g., diabetes, hypertension, and obesity; (Wolkove et al., 2007; Neikrug and Ancoli-Israel, 2010; Beccuti and Pannain, 2011; Lyons et al., 2015), psychiatric disorders (e.g., depression and psychoses; Wolkove et al., 2007; Kaufmann et al., 2011), neurological diagnoses (e.g., AD, dementia, Parkinson's Disease; Wolkove et al., 2007; Neikrug and Ancoli-Israel, 2010), cancer (Neikrug and Ancoli-Israel, 2010), and alcohol consumption (Wolkove et al., 2007; Neikrug and Ancoli-Israel, 2010). In addition, our results indicated an increase in the average number of health conditions over the last decade. Furthermore, our results highlighted that many co-morbidities (depression, cardiovascular risk factors, and neurological disorders) steadily increased over time, which supports a coupling linear trend between these medical conditions and sleep disturbances. These findings further support the proposed rationale that the increased number of health conditions and increased rates of particular health conditions (e.g., obesity) in the last decade is associated with the increased rates in sleep disturbances over time (Kronholm et al., 2008; Pallesen et al., 2014; Ford et al., 2015). The coupled increase trend in sleep disturbances and medical conditions may also account for the increasing in-hospital costs.

Hospital characteristics differed across the three classes of sleep disturbance. Rates for insomnia and OSD were higher in investor-owned hospitals, hospitals located in rural areas, and hospitals with smaller bed sizes. In contrast, rates for OSA were higher in non-profit hospitals, hospitals located in urban areas, and hospitals with larger bed sizes. There is only limited research on these differences in hospital characteristics as it relates to various types of sleep disturbances. It is possible that neighborhood characteristics (e.g., noise pollution, lighting, and air quality in neighborhoods near hospital) and individual characteristics (e.g., proportion of individuals below the poverty level residing near hospital) may explain these differences. Further research is warranted to understand how some of these modifiable factors may reduce the number of individuals with sleep disturbances, and the subsequent hospital costs associated with sleep disturbances.

Despite many of study strengths including national representativeness, large sample size, and availability of extensive healthcare data that allow for trends analyses, the current study is not without limitations. First, it relied on administrative database using ICD-9-CM codes. These codes may not be categorized based upon the standard diagnosis criteria for sleep disorders, which may lead to diagnosis misclassification. However, the AHRQ periodically ensures quality checks with internal and external validation. Second, discharge abstracts are de-identified, thus precluding longitudinal analyses. Third, the structure of NIS limits our ability to detect multiple admissions and/or discharges from the same condition per patient, including those with insomnia. In addition, detailed patient data are lacking such that individual medication regimens and laboratory results are missing. This precludes examining important covariates we would otherwise have included. Older adults are likely taking a number of medications. Previous research has suggested that this polypharmacy patient profile is even more prominent amongst older individuals suffering from sleep (Neikrug and Ancoli-Israel, 2010). Future research is needed to explore how the relationship between sleep disturbances and hospitalization characteristics/outcomes is explained by the number and extent of an individual's medication regimen. Fourth, NIS is limited to hospitalized patients with sleep disturbance and thus trend results may not be similar in the community. Nonetheless, sleep disturbances are often underdiagnosed and untreated, particularly within the older adult population (Groth, 2005; Wolkove et al., 2007; Neikrug and Ancoli-Israel, 2010) and thus our study's estimated rates are likely conservative. As with any retrospective administrative data analysis, there is potential for bias from missing data; however, it is unlikely that missing data will have a large effect on the results because of the large sample size of the current study. Furthermore, we were unable to compare frequencies of hospitalization between patients with and without sleep disturbance over pre-set periods of time (e.g., a month or a year).

This study observed that while rates of sleep disturbances are increasing within hospital settings, the relatively low rates may reflect that many older adults' sleep disorders remain underdiagnosed. Since older adults' sleep disturbances are associated with an increase in healthcare costs, educational interventions designed to train healthcare professionals in recognizing and effectively treating sleep disturbances may assist in reducing these healthcare expenditures.

## Author contributions

AG: Study conception, literature search and review, plan of analysis, interpretation of findings, write-up of parts of the manuscript, revision of the manuscript. MB: Study conception, plan of analysis, data management, statistical analysis, interpretation of findings, write-up of parts of the manuscript, revision of the manuscript. HB: Plan of analysis, literature search, write-up of parts of the manuscript, revision of the manuscript. HL: Literature search and review, data management, revision of the manuscript. RS: Interpretation of findings, write-up of parts of the manuscript, revision of the manuscript. AZ: Plan of analysis, interpretation of findings, revision of the manuscript. CG: Literature search and review, interpretation of findings, write-up of parts of the manuscript, revision of the manuscript. SE: Plan of analysis, data management, data acquisition, interpretation of findings, revision of the manuscript.

### Conflict of interest statement

The authors declare that the research was conducted in the absence of any commercial or financial relationships that could be construed as a potential conflict of interest.

## Footnotes

^1^Healthcare Cost and Utilization Project (HCUP), Nationwide Inpatient Sample (NIS) Documentation. Available online at: http://www.hcup-us.ahrq.gov/db/nation/nis/nisdbdocumentation.jsp (Accessed February 5, 2015).

## Abbreviations

AHRQ: Agency for Healthcare Research and Quality
AIDS: Acquired Immune Deficiency Syndrome
CHF: Congestive Heart Failure
CI: Confidence Interval
HCUP: Healthcare Cost and Utilization Project
ICD-9-CM: International Classification of Diseases, Ninth Revision, Clinical Modification
LOS: length of stay
MR: Mortality risk
NIS: Nationwide Inpatient Sample
OR: Odds Ratio
OSA: Obstructive Sleep Apnea
OSD: Other sleep disturbances
SE: Standard Error
SID: State Inpatient Databases
TC: total charges.
