# Supplementary material for: Sleep Disturbances among Older Adults in the United States, 2002–2012: Nationwide Inpatient Rates, Predictors, and Outcomes
Source: Front Aging Neurosci. 2016 Nov 15;8:266. doi: 10.3389/fnagi.2016.00266 (PMC5109617; doi:10.3389/fnagi.2016.00266)
Supplement: Supplementary file 1 [file DataSheet1.docx]

**APPENDIX I**

NIS contains clinical and nonclinical data elements for each hospital stay, including: Primary and secondary diagnoses and procedures; patient demographic characteristics (e.g., sex, age, race, median household income for ZIP Code); hospital characteristics (e.g., ownership); expected payment source; TC; discharge status; LOS; severity and comorbidity measures. Many of these variables were included in the NIS core file which contains discharge-level data along with AHA hospital file which contain hospital characteristics and weights data.

Noteworthy changes occurred to the sampling design of NIS as of 1998, which included the following: (1) Revising definitions of the strata variables; (2) Excluding rehabilitation hospitals from the NIS hospital universe; (3) Changing the calculation of hospital universe discharges for the weights. Between 1998 and 2002, the number of hospitals included in the sample covered 13 more States, thus going from a 22-State to a 35-State geographical coverage. The initial number of States included in 1988 was limited to 8. After 2002, no new States were added.

2012 NIS was redesigned to improve national estimates, making them more precise. Three major changes were made to this wave forward: (a) Sample design was modified so that the NIS would be a sample of discharge records from all HCUP-participating hospitals, rather than sampling the hospitals themselves from which all discharges would be retained; (b) The 2012 NIS forward uses the definitions of hospitals and discharges supplied by the statewide data organizations that contribute to HCUP, as opposed to definitions used by the AHA Annual Survey; (c) The 2012 NIS forward removed State and hospital identifiers and other data elements to enhance confidentiality.

**APPENDIX II**

The table below illustrates the International Classification of Diseases, Ninth Revision (ICD-9) codes used for insomnia, obstructive sleep apnea (OSA), and other sleep disturbances (OSD). ICD-9 codes were retrieved for the Centers of Medicare & Medicaid Services (Services, 2015). The ICD-9 codes representative of insomnia have been used in the previous literature (Hermes and Rosenheck, 2014).

**Table S1.** Sex-specific and overall time trends in insomnia, obstructive sleep apnea (OSA) and other sleep disturbances (OSD), (as any diagnosis) rates (%) in the inpatient older adult population (60+y); NIS, 2002 to 2012

|  |  |  | **Rates of sleep disturbances (Wt %±SE)** | | | | | | | | | | |  |
| --- | --- | --- | --- | --- | --- | --- | --- | --- | --- | --- | --- | --- | --- | --- |
|  |  | **Unwt. N, 60+y,**  **2002-2012** | **2002** | **2003** | **2004** | **2005** | **2006** | **2007** | **2008** | **2009** | **2010** | **2011** | **2012** |  |
| **Men and women** |  |  |  |  |  |  |  |  |  |  |  |  |  |  |
| P<0.001, ↑ | Insomnia | 34,988,434 | 0.27±0.00 | 0.31±0.00 | 0.38±0.00 | 0.50±0.00 | 0.60±0.00 | 0.73±0.00 | 0.85±0.00 | 0.97±0.00 | 1.04±0.00 | 1.22±0.00 | 1.29±0.03 |  |
| P<0.001, ↑ | OSA | 34,988,500 | 0.00±0.00 | 0.00±0.00 | 0.00±0.00 | 0.21±0.01 | 1.47±0.05 | 2.09±0.06 | 2.71±0.07 | 3.36±0.08 | 3.93±0.10 | 4.48±0.10 | 5.01±0.05 |  |
| P<0.001, ↑ | OSD | 34,988,407 | 0.06±0.00 | 0.04±0.01 | 0.04±0.00 | 0.05±0.04 | 0.06±0.00 | 0.06±0.00 | 0.07±0.00 | 0.07±0.00 | 0.07±0.00 | 0.08±0.00 | 0.08±0.00 |  |
| **Men** |  |  |  |  |  |  |  |  |  |  |  |  |  |  |
| P<0.001, ↑ | Insomnia | 15,374,017 | 0.23±0.00 | 0.26±0.00 | 0.31±0.00 | 0.40±0.00 | 0.49±0.00 | 0.59±0.00 | 0.69±0.00 | 0.78±0.00 | 0.84±0.00 | 1.02±0.00 | 1.10±0.00 |  |
| P<0.001, ↑ | OSA | 15,374,065 | 0.00±0.00 | 0.00±0.00 | 0.00±0.00 | 0.29±0.01 | 1.96±0.07 | 2.74±0.08 | 3.56±0.09 | 4.34±0.11 | 5.04±0.12 | 5.72±0.13 | 6.33±0.07 |  |
| P<0.001, ↑ | OSD | 15,374,006 | 0.04±0.00 | 0.04±0.00 | 0.05±0.00 | 0.05±0.00 | 0.05±0.00 | 0.06±0.00 | 0.07±0.00 | 0.06±0.00 | 0.06±0.00 | 0.07±0.00 | 0.08±0.00 |  |
| **Women** |  |  |  |  |  |  |  |  |  |  |  |  |  |  |
| P<0.001, ↑ | Insomnia | 19,614,417 | 0.30±0.01 | 0.35±0.01 | 0.44±0.01 | 0.58±0.02 | 0.68±0.02 | 0.84±0.00 | 0.98±0.03 | 1.11±0.03 | 1.18±0.04 | 1.38±0.03 | 1.46±0.02 |  |
| P<0.001, ↑ | OSA | 19,614,435 | 0.00±0.00 | 0.00±0.00 | 0.00±0.00 | 0.15±0.01 | 1.09±0.04 | 1.57±0.05 | 2.05±0.06 | 2.57±0.06 | 3.04±0.08 | 3.49±0.09 | 3.92±0.04 |  |
| P<0.001, ↑ | OSD | 19,614,401 | 0.03±0.01 | 0.04±0.00 | 0.05±0.00 | 0.05±0.00 | 0.06±0.00 | 0.06±0.00 | 0.07±0.00 | 0.07±0.00 | 0.08±0.00 | 0.09±0.00 | 0.09±0.00 |  |

**Table S2.** Sex- and age-specific time trends in insomnia, obstructive sleep apnea (OSA) and other sleep disturbances (OSD), (as any diagnosis) rates (%) in the inpatient older adult population (60+y); NIS, 2002 to 2012

|  |  |  | **Rates of sleep disturbances (Wt %±SE)** | | | | | | | | | | |  |
| --- | --- | --- | --- | --- | --- | --- | --- | --- | --- | --- | --- | --- | --- | --- |
|  |  | **Unwt. N, 60+y,**  **2002-2012** | **2002** | **2003** | **2004** | **2005** | **2006** | **2007** | **2008** | **2009** | **2010** | **2011** | **2012** |  |
| **Men** |  |  |  |  |  |  |  |  |  |  |  |  |  |  |
|  |  |  |  |  |  |  |  |  |  |  |  |  |  |  |
| 60-64 |  |  |  |  |  |  |  |  |  |  |  |  |  |  |
| P<0.001, ↑ | Insomnia | 2,645,262 | 0.21±0.01 | 0.25±0.01 | 0.33±0.01 | 0.42±0.00 | 0.49±0.00 | 0.62±0.00 | 0.74±0.00 | 0.79±0.00 | 0.90±0.00 | 1.11±0.00 | 1.25±0.00 |  |
| P<0.001, ↑ | OSA | 2,645,281 | 0.00±0.00 | 0.00±0.00 | 0.00±0.00 | 0.51±0.02 | 3.17±0.01 | 4.24±0.01 | 5.37±0.01 | 6.30±0.15 | 7.10±0.19 | 7.84±0.18 | 8.19±0.10 |  |
|  | OSD | 2,645,259 | 0.06±0.01 | 0.05±0.00 | 0.06±0.01 | 0.05±0.01 | 0.05±0.00 | 0.06±0.01 | 0.08±0.01 | 0.07±0.01 | 0.07±0.01 | 0.07±0.01 | 0.09±0.00 |  |
| 65-69 |  |  |  |  |  |  |  |  |  |  |  |  |  |  |
| P<0.001, ↑ | Insomnia | 2,750,499 | 0.25±0.01 | 0.26±0.00 | 0.30±0.01 | 0.41±0.02 | 0.49±0.02 | 0.60±0.00 | 0.71±0.00 | 0.79±0.00 | 0.87±0.00 | 1.06±0.00 | 1.14±0.00 |  |
| P<0.001, ↑ | OSA | 2,750,510 | 0.00±0.00 | 0.00±0.00 | 0.00±0.00 | 0.43±0.02 | 2.79±0.10 | 3.83±0.12 | 5.00±0.13 | 5.95±0.14 | 6.74±0.17 | 7.56±0.17 | 8.38±0.10 |  |
| P<0.001, ↑ | OSD | 2,750,497 | 0.05±0.01 | 0.04±0.00 | 0.06±0.00 | 0.05±0.00 | 0.07±0.00 | 0.06±0.01 | 0.07±0.01 | 0.07±0.01 | 0.06±0.01 | 0.09±0.01 | 0.08±0.01 |  |
| 70-74 |  |  |  |  |  |  |  |  |  |  |  |  |  |  |
| P<0.001, ↑ | Insomnia | 2,700,491 | 0.23±0.01 | 0.26±0.01 | 0.31±0.01 | 0.37±0.01 | 0.49±0.02 | 0.57±0.02 | 0.68±0.03 | 0.77±0.03 | 0.84±0.03 | 1.03±0.03 | 1.10±0.02 |  |
| P<0.001, ↑ | OSA | 2,700,502 | 0.00±0.00 | 0.00±0.00 | 0.00±0.00 | 0.35±0.02 | 2.26±0.08 | 3.17±0.01 | 4.18±0.11 | 5.06±0.13 | 5.94±0.15 | 6.78±0.16 | 7.59±0.10 |  |
| P<0.001, ↑ | OSD | 2,700,490 | 0.04±0.01 | 0.04±0.00 | 0.05±0.01 | 0.05±0.01 | 0.06±0.00 | 0.06±0.00 | 0.07±0.01 | 0.06±0.01 | 0.07±0.01 | 0.07±0.01 | 0.09±0.01 |  |
| 75-79 |  |  |  |  |  |  |  |  |  |  |  |  |  |  |
| P<0.001, ↑ | Insomnia | 2,712,444 | 0.25±0.01 | 0.26±0.01 | 0.30±0.01 | 0.40±0.02 | 0.50±0.02 | 0.56±0.02 | 0.68±0.02 | 0.74±0.03 | 0.81±0.03 | 0.99±0.03 | 1.04±0.00 |  |
| P<0.001, ↑ | OSA | 2,712,449 | 0.00±0.00 | 0.00±0.00 | 0.00±0.00 | 0.24±0.01 | 1.70±0.07 | 2.43±0.08 | 3.14±0.09 | 3.84±0.12 | 4.63±0.13 | 5.34±0.14 | 6.00±0.08 |  |
| P<0.001, ↑ | OSD | 2,712,443 | 0.04±0.00 | 0.05±0.01 | 0.05±0.00 | 0.05±0.00 | 0.05±0.01 | 0.05±0.01 | 0.06±0.00 | 0.06±0.01 | 0.06±0.01 | 0.08±0.01 | 0.08±0.01 |  |
| 80-84 |  |  |  |  |  |  |  |  |  |  |  |  |  |  |
| P<0.001, ↑ | Insomnia | 2,346,769 | 0.22±0.01 | 0.26±0.01 | 0.32±0.01 | 0.39±0.02 | 0.45±0.00 | 0.57±0.02 | 0.65±0.03 | 0.82±0.03 | 0.84±0.03 | 0.97±0.03 | 1.04±0.03 |  |
| P<0.001, ↑ | OSA | 2,346,771 | 0.00±0.00 | 0.00±0.00 | 0.00±0.00 | 0.15±0.01 | 1.06±0.05 | 1.60±0.06 | 2.01±0.07 | 2.76±0.09 | 3.24±0.10 | 3.90±0.10 | 4.38±0.07 |  |
| P<0.001, ↑ | OSD | 2,346,767 | 0.03±0.01 | 0.03±0.00 | 0.04±0.01 | 0.06±0.01 | 0.05±0.00 | 0.05±0.00 | 0.06±0.00 | 0.06±0.00 | 0.06±0.00 | 0.07±0.00 | 0.06±0.00 |  |
| 85+ |  |  |  |  |  |  |  |  |  |  |  |  |  |  |
| P<0.001, ↑ | Insomnia | 2,214,312 | 0.25±0.02 | 0.27±0.02 | 0.31±0.02 | 0.43±0.02 | 0.54±0.02 | 0.58±0.02 | 0.68±0.03 | 0.78±0.03 | 0.82±0.04 | 0.97±0.03 | 0.97±0.02 |  |
| P<0.001, ↑ | OSA | 2,214,312 | 0.00±0.00 | 0.00±0.00 | 0.00±0.00 | 0.07±0.01 | 0.49±0.02 | 0.74±0.03 | 0.99±0.03 | 1.34±0.05 | 1.64±0.06 | 1.97±0.06 | 2.30±0.04 |  |
| P<0.001, ↑ | OSD | 2,214,310 | 0.03±0.00 | 0.03±0.00 | 0.04±0.00 | 0.05±0.00 | 0.05±0.00 | 0.06±0.00 | 0.06±0.01 | 0.06±0.01 | 0.05±0.01 | 0.06±0.01 | 0.07±0.00 |  |
| **Women** |  |  |  |  |  |  |  |  |  |  |  |  |  |  |
| 60-64 |  |  |  |  |  |  |  |  |  |  |  |  |  |  |
| P<0.001, ↑ | Insomnia | 2,651,816 | 0.30±0.02 | 0.35±0.02 | 0.48±0.02 | 0.63±0.03 | 0.74±0.03 | 0.89±0.03 | 1.09±0.04 | 1.18±0.04 | 1.28±0.04 | 1.60±0.05 | 1.71±0.03 |  |
| P<0.001, ↑ | OSA | 2,651,829 | 0.00±0.00 | 0.00±0.00 | 0.00±0.00 | 0.34±0.02 | 2.36±0.08 | 3.24±0.10 | 4.09±0.11 | 4.95±0.11 | 5.72±0.15 | 6.22±0.14 | 6.81±0.08 |  |
| P<0.001, ↑ | OSD | 2,651,817 | 0.04±0.01 | 0.05±0.00 | 0.07±0.01 | 0.07±0.01 | 0.08±0.01 | 0.08±0.01 | 0.10±0.01 | 0.10±0.01 | 0.10±0.01 | 0.10±0.01 | 0.11±0.01 |  |
|  |  |  |  |  |  |  |  |  |  |  |  |  |  |  |
| 65-69 |  |  |  |  |  |  |  |  |  |  |  |  |  |  |
| P<0.001, ↑ | Insomnia | 2,857,255 | 0.33±0.02 | 0.37±0.02 | 0.45±0.02 | 0.53±0.02 | 0.71±0.03 | 0.90±0.03 | 1.06±0.04 | 1.18±0.04 | 1.31±0.05 | 1.52±0.05 | 1.58±0.03 |  |
| P<0.001, ↑ | OSA | 2,857,256 | 0.00±0.00 | 0.00±0.00 | 0.00±0.00 | 0.25±0.01 | 1.89±0.01 | 2.67±0.08 | 3.42±0.10 | 4.25±0.11 | 4.83±0.12 | 5.49±0.14 | 6.15±0.08 |  |
| P<0.001, ↑ | OSD | 2,857,248 | 0.04±0.01 | 0.05±0.01 | 0.06±0.01 | 0.06±0.01 | 0.08±0.01 | 0.06±0.01 | 0.08±0.01 | 0.09±0.01 | 0.09±0.01 | 0.10±0.01 | 0.10±0.01 |  |
| 70-74 |  |  |  |  |  |  |  |  |  |  |  |  |  |  |
| P<0.001, ↑ | Insomnia | 3,012,206 | 0.31±0.02 | 0.38±0.02 | 0.45±0.02 | 0.58±0.02 | 0.70±0.03 | 0.84±0.03 | 1.02±0.04 | 1.19±0.04 | 1.21±0.04 | 1.44±0.04 | 1.51±0.03 |  |
| P<0.001, ↑ | OSA | 3,012,206 | 0.00±0.00 | 0.00±0.00 | 0.00±0.00 | 0.19±0.01 | 1.40±0.06 | 1.97±0.07 | 2.64±0.08 | 3.19±0.08 | 3.89±0.11 | 4.50±0.12 | 4.97±0.07 |  |
| P<0.001, ↑ | OSD | 3,012,203 | 0.04±0.01 | 0.04±0.00 | 0.06±0.00 | 0.06±0.00 | 0.07±0.01 | 0.07±0.01 | 0.08±0.01 | 0.09±0.01 | 0.09±0.01 | 0.08±0.01 | 0.09±0.01 |  |
| 75-79 |  |  |  |  |  |  |  |  |  |  |  |  |  |  |
| P<0.001, ↑ | Insomnia | 3,348,033 | 0.29±0.01 | 0.36±0.02 | 0.44±0.02 | 0.58±0.00 | 0.67±0.03 | 0.87±0.00 | 0.97±0.04 | 1.11±0.04 | 1.17±0.04 | 1.37±0.04 | 1.49±0.03 |  |
| P<0.001, ↑ | OSA | 3,348,038 | 0.00±0.00 | 0.00±0.00 | 0.00±0.00 | 0.12±0.01 | 0.90±0.04 | 1.39±0.05 | 1.78±0.06 | 2.32±0.07 | 2.74±0.08 | 3.26±0.09 | 3.61±0.05 |  |
| P<0.001, ↑ | OSD | 3,348,032 | 0.03±0.00 | 0.04±0.00 | 0.05±0.00 | 0.05±0.00 | 0.06±0.00 | 0.06±0.01 | 0.08±0.01 | 0.07±0.01 | 0.07±0.01 | 0.09±0.01 | 0.08±0.01 |  |
| 80-84 |  |  |  |  |  |  |  |  |  |  |  |  |  |  |
| P<0.001, ↑ | Insomnia | 3,366,634 | 0.31±0.02 | 0.35±0.01 | 0.42±0.02 | 0.58±0.02 | 0.67±0.03 | 0.81±0.03 | 0.93±0.03 | 1.07±0.04 | 1.15±0.04 | 1.24±0.04 | 1.33±0.03 |  |
| P<0.001, ↑ | OSA | 3,366,636 | 0.00±0.00 | 0.00±0.00 | 0.00±0.00 | 0.07±0.01 | 0.53±0.02 | 0.78±0.03 | 1.13±0.04 | 1.39±0.04 | 1.71±0.06 | 2.09±0.06 | 2.36±0.04 |  |
| P<0.001, ↑ | OSD | 3,366,633 | 0.04±0.01 | 0.04±0.00 | 0.04±0.01 | 0.05±0.00 | 0.06±0.00 | 0.06±0.01 | 0.06±0.01 | 0.05±0.00 | 0.07±0.01 | 0.07±0.01 | 0.07±0.01 |  |
| 85+ |  |  |  |  |  |  |  |  |  |  |  |  |  |  |
| P<0.001, ↑ | Insomnia | 4,373,237 | 0.28±0.02 | 0.33±0.01 | 0.39±0.02 | 0.53±0.02 | 0.61±0.02 | 0.77±0.03 | 0.86±0.03 | 1.10±0.04 | 1.06±0.04 | 1.23±0.04 | 1.24±0.02 |  |
| P<0.001, ↑ | OSA | 4,373,234 | 0.00±0.00 | 0.00±0.00 | 0.00±0.00 | 0.03±0.00 | 0.23±0.01 | 0.31±0.01 | 0.44±0.02 | 0.58±0.02 | 0.73±0.03 | 0.91±0.03 | 1.04±0.02 |  |
| P<0.001, ↑ | OSD | 4,373,232 | 0.02±0.00 | 0.03±0.00 | 0.05±0.01 | 0.04±0.00 | 0.04±0.00 | 0.06±0.01 | 0.06±0.00 | 0.06±0.00 | 0.06±0.00 | 0.07±0.00 | 0.06±0.00 |  |

↑Increasing linear trend.

*Abbreviations*: NIS=Nationwide Inpatient Sample; OSA=Obstructive Sleep Apnea; OSD=Other Sleep Disturbances.

**Table S3.** Trends in co-morbidity rates (%) among insomnia, OSA and OSD patients; NIS, 2002 to 2012

|  |  | **Rates of co-morbidity (Wt %±SE)** | | | | | | | | | | |
| --- | --- | --- | --- | --- | --- | --- | --- | --- | --- | --- | --- | --- |
|  | **P-trend** | **2002** | **2003** | **2004** | **2005** | **2006** | **2007** | **2008** | **2009** | **2010** | **2011** | **2012** |
| **%±SE** |  |  |  |  |  |  |  |  |  |  |  |  |
| **AIDS** |  |  |  |  |  |  |  |  |  |  |  |  |
| Insomnia | P<0.01, ↑ | . ± . | 0.03±0.01 | 0.02±0.01 | 0.03±0.01 | 0.03±0.01 | 0.03±0.01 | 0.04±0.02 | 0.05±0.02 | 0.05±0.02 | 0.07±0.02 | 0.05±0.01 |
| OSA | P<0.05, ↑ | . ± . | . ± . | . ± . | 0.02±0.02 | 0.01±0.00 | 0.01±0.00 | 0.03±0.01 | 0.03±0.01 | 0.03±01 | 0.02±0.01 | 0.03±0.00 |
| OSD | P<0.01, ↑ | . ± . | . ± . | . ± . | . ± . | . ± . | . ± . | . ± . | . ± . | . ± . | 0.07±0.05 | 0.04±0.04 |
| **Alcohol Abuse** |  |  |  |  |  |  |  |  |  |  |  |  |
| Insomnia | P<0.001, ↑ | 2.16±0.19 | 1.88±0.16 | 1.93±0.15 | 1.99±0.12 | 2.33±0.12 | 2.42±0.13 | 2.59±0.13 | 2.62±0.11 | 2.98±0.13 | 3.11±0.11 | 3.32±0.10 |
| OSA | P<0.001, ↑ | . ± . | . ± . | . ± . | 1.71±0.16 | 1.33±0.07 | 1.46±0.06 | 1.63±0.08 | 1.52±0.06 | 1.77±0.07 | 1.82±0.05 | 1.82±0.04 |
| OSD | P<0.01, ↑ | 2.42±0.68 | 1.89±0.37 | 1.55±0.35 | 1.38±0.29 | 2.46±0.42 | 2.15±0.39 | 2.03±0.37 | 2.07±0.32 | 2.95±0.38 | 2.60±0.33 | 2.85±0.34 |
| **Def. anemia** |  |  |  |  |  |  |  |  |  |  |  |  |
| Insomnia | P<0.001, ↑ | 14.43±0.47 | 14.95±0.52 | 15.92±0.54 | 17.09±0.43 | 18.00±0.46 | 19.35±0.49 | 21.35±0.49 | 22.21±0.38 | 21.76±0.44 | 22.54±0.40 | 21.13±0.26 |
| OSA | P<0.001, ↑ | . ± . | . ± . | . ± . | 14.21±0.53 | 15.61±0.49 | 17.20±0.41 | 19.81±0.46 | 19.60±0.42 | 20.00±0.43 | 21.36±0.33 | 20.74±0.19 |
| OSD | P<0.001, ↑ | 9.35±1.77 | 11.57±0.97 | 12.06±0.94 | 14.49±0.97 | 13.90±0.92 | 16.97±0.98 | 16.92±0.98 | 18.55±0.90 | 19.16±0.94 | 20.57±1.04 | 18.48±0.85 |
| **Rh. Arthritis** |  |  |  |  |  |  |  |  |  |  |  |  |
| Insomnia | P<0.001, ↑ | 2.86±0.20 | 3.19±0.19 | 2.98±0.17 | 3.02±0.16 | 3.01±0.15 | 3.65±0.15 | 3.65±0.15 | 3.77±0.13 | 4.05±0.15 | 4.06±0.12 | 4.03±0.11 |
| OSA | P<0.001, ↑ | . ± . | . ± . | . ± . | 3.02±0.21 | 2.82±0.11 | 2.90±0.09 | 3.16±0.09 | 3.51±0.08 | 3.64±0.10 | 3.89±0.08 | 3.97±0.06 |
| OSD | P<0.001, ↑ | 2.49±0.41 | 2.37±045 | 3.73±0.49 | 2.32±0.40 | 3.22±0.43 | 3.13±0.41 | 3.07±0.46 | 3.60±0.42 | 3.65±0.46 | 4.54±0.43 | 4.38±0.40 |
| **Blood loss anemia** |  |  |  |  |  |  |  |  |  |  |  |  |
| Insomnia | P<0.001, ↑ | 1.40±0.13 | 1.45±0.13 | 1.75±0.14 | 1.71±0.14 | 1.93±0.12 | 1.69±0.11 | 1.55±0.09 | 1.47±0.11 | 1.44±0.14 | 1.19±0.07 | 1.16±0.07 |
| OSA | P<0.001, ↓ | . ± . | . ± . | . ± . | 1.62±0.18 | 1.44±0.08 | 1.50±0.07 | 1.36±0.07 | 1.18±0.06 | 1.22±0.06 | 1.12±0.04 | 1.02±0.03 |
| OSD | P>0.05 | 0.66±0.29 | 0.79±0.24 | 1.01±0.26 | 1.01±0.27 | 1.73±0.32 | 1.73±0.31 | 1.05±0.26 | 1.11±0.23 | 0.99±0.21 | 1.14±0.24 | 1.12±0.22 |
| **CHF** |  |  |  |  |  |  |  |  |  |  |  |  |
| Insomnia | P<0.001, ↓ | 10.22±0.40 | 10.49±0.43 | 10.35±0.36 | 10.96±0.32 | 11.09±0.37 | 10.59±0.27 | 10.22±0.29 | 10.27±0.25 | 9.78±0.26 | 9.92±0.23 | 9.70±0.16 |
| OSA | P>0.05 | . ± . | . ± . | . ± . | 19.59±0.62 | 19.92±0.44 | 20.12±0.40 | 19.40±0.42 | 19.21±0.36 | 19.04±0.36 | 19.95±0.28 | 19.49±0.17 |
| OSD | P>0.05 | 10.22±1.82 | 10.93±0.87 | 11.13±1.06 | 11.12±0.88 | 11.02±0.85 | 10.33±0.74 | 10.32±0.73 | 10.19±0.80 | 10.12±0.75 | 10.52±0.71 | 10.85±0.62 |
| **Chronic pulmonary** |  |  |  |  |  |  |  |  |  |  |  |  |
| Insomnia | P<0.01, ↑ | 22.04±0.66 | 21.61±0.56 | 21.97±0.49 | 23.34±0.47 | 24.25±0.45 | 23.77±0.40 | 23.11±0.43 | 23.49±0.35 | 23.19±0.37 | 23.58±0.33 | 23.61±0.25 |
| OSA | P<0.001, ↓ | . ± . | . ± . | . ± . | 39.13±0.78 | 40.66±0.64 | 40.49±0.55 | 38.32±0.53 | 37.76±0.45 | 37.15±0.48 | 37.90±0.39 | 37.30±0.22 |
| OSD | P<0.05, ↑ | 18.65±2.34 | 23.82±1.31 | 22.27±1.35 | 24.96±1.37 | 25.38±1.30 | 25.22±1.13 | 22.55±1.08 | 23.12±1.04 | 25.56±1.08 | 24.57±0.90 | 25.31±0.89 |
| **Coagulopathy** |  |  |  |  |  |  |  |  |  |  |  |  |
| Insomnia | P<0.001, ↑ | 1.54±0.14 | 1.98±0.14 | 1.85±0.13 | 1.84±0.13 | 2.18±0.13 | 2.46±0.15 | 2.62±0.12 | 3.30±0.13 | 3.80±0.16 | 3.94±0.12 | 4.20±0.11 |
| OSA | P<0.001, ↑ | . ± . | . ± . | . ± . | 2.52±0.22 | 2.61±0.11 | 2.99±0.11 | 3.40±0.10 | 3.76±0.12 | 4.53±0.14 | 4.84±0.11 | 5.11±0.08 |
| OSD | P<0.001, ↑ | 0.94±0.32 | 0.92±0.25 | 1.71±0.33 | 2.14±0.36 | 1.13±0.24 | 2.17±0.31 | 2.92±0.38 | 1.96±0.33 | 3.56±0.50 | 4.41±0.37 | 3.46±0.38 |
| **Depression** |  |  |  |  |  |  |  |  |  |  |  |  |
| Insomnia | P<0.001, ↑ | 19.94±0.53 | 20.83±0.51 | 21.53±0.48 | 22.18±0.44 | 23.92±0.50 | 24.55±0.40 | 25.80±0.46 | 25.92±0.39 | 27.22±0.52 | 27.43±0.43 | 27.25±0.26 |
| OSA | P<0.001, ↑ | . ± . | . ± . | . ± . | 10.18±0.46 | 11.58±0.35 | 12.50±0.30 | 14.24±0.38 | 13.69±0.28 | 15.49±0.33 | 16.34±0.30 | 16.48±0.16 |
| OSD | P<0.001, ↑ | 13.39±2.32 | 18.08±1.29 | 18.71±1.17 | 17.11±1.19 | 18.27±1.05 | 20.67±1.17 | 22.37±1.14 | 22.38±1.04 | 22.19±1.01 | 22.04±0.97 | 23.62±0.84 |
| **Diabetes, uncomp.** |  |  |  |  |  |  |  |  |  |  |  |  |
| Insomnia | P<0.001, ↑ | 15.33±0.52 | 15.69±0.44 | 16.61±0.41 | 17.22±0.38 | 17.90±0.38 | 18.36±0.34 | 18.57±0.34 | 19.77±0.32 | 19.75±0.39 | 19.84±0.31 | 20.58±0.24 |
| OSA | P<0.001, ↑ | . ± . | . ± . | . ± . | 36.22±0.71 | 38.01±0.41 | 38.83±0.41 | 38.67±0.41 | 39.80±0.41 | 39.52±0.40 | 40.29±0.32 | 39.95±0.20 |
| OSD | P<0.001, ↑ | 17.43±1.80 | 17.20±1.18 | 19.10±1.21 | 20.36±1.13 | 20.28±1.02 | 20.00±1.13 | 22.16±1.01 | 20.64±0.94 | 23.00±1.04 | 21.50±0.93 | 22.78±0.81 |
| **Diabetes, comp.** |  |  |  |  |  |  |  |  |  |  |  |  |
| Insomnia | P<0.001, ↑ | 2.88±0.23 | 2.90±0.22 | 2.70±0.17 | 3.09±0.16 | 3.29±0.17 | 3.61±0.15 | 3.56±0.16 | 3.77±0.16 | 3.77±0.16 | 4.21±0.18 | 3.95±0.11 |
| OSA | P<0.001, ↑ | . ± . | . ± . | . ± . | 7.89±0.44 | 8.89±0.32 | 9.23±0.24 | 9.68±0.30 | 9.16±0.21 | 9.56±0.25 | 9.91±0.24 | 9.95±0.12 |
| OSD | P>0.05 | 2.69±0.69 | 3.49±0.51 | 3.57±0.54 | 3.92±0.58 | 3.59±0.64 | 4.73±0.51 | 4.70±0.53 | 4.20±0.47 | 3.64±0.45 | 4.14±0.45 | 4.42±0.43 |
| **Drug abuse** |  |  |  |  |  |  |  |  |  |  |  |  |
| Insomnia | P<0.001, ↑ | 0.91±0.11 | 0.89±0.10 | 0.93±0.11 | 1.06±0.09 | 0.90±0.08 | 1.04±0.07 | 1.17±0.09 | 1.35±0.09 | 1.57±0.11 | 1.69±0.08 | 1.83±0.08 |
| OSA | P<0.001, ↑ | . ± . | . ± . | . ± . | 0.42±0.09 | 0.34±0.03 | 0.39±0.03 | 0.56±0.03 | 0.54±0.03 | 0.70±0.04 | 0.83±0.05 | 0.86±0.03 |
| OSD | P<0.001, ↑ | 0.50±0.22 | 1.08±0.31 | 0.63±0.20 | 2.54±0.38 | 6.08±0.63 | 7.50±0.68 | 7.14±0.71 | 7.64±0.68 | 7.73±0.78 | 6.59±0.54 | 7.47±0.55 |
| **Hypertension** |  |  |  |  |  |  |  |  |  |  |  |  |
| Insomnia | P<0.001, ↑ | 52.38±0.64 | 57.94±0.65 | 59.84±0.59 | 61.51±0.49 | 62.74±0.55 | 64.62±0.46 | 66.83±0.49 | 67.83±0.41 | 68.67±0.40 | 68.66±0.35 | 69.74±0.26 |
| OSA | P<0.001, ↑ | . ± . | . ± . | . ± . | 66.49±0.71 | 69.05±0.49 | 69.28±0.39 | 72.46±0.41 | 72.68±0.36 | 74.36±0.37 | 75.27±0.26 | 76.26±0.15 |
| OSD | P<0.001, ↑ | 46.36±2.52 | 54.56±1.83 | 56.47±1.49 | 57.02±1.35 | 61.16±1.27 | 61.98±1.27 | 64.90±1.18 | 63.83±1.19 | 68.32±1.16 | 68.01±0.95 | 68.34±0.98 |
| **Hypothyroidism** |  |  |  |  |  |  |  |  |  |  |  |  |
| Insomnia | P<0.001, ↑ | 13.55±0.48 | 14.16±0.41 | 14.39±0.40 | 15.34±0.35 | 16.43±0.36 | 17.05±0.34 | 17.76±0.36 | 18.90±0.28 | 18.46±0.31 | 19.44±0.26 | 19.86±0.21 |
| OSA | P<0.001, ↑ | . ± . | . ± . | . ± . | 11.92±0.43 | 13.15±0.28 | 14.19±0.24 | 15.47±0.26 | 15.81±0.22 | 16.39±0.22 | 17.55±0.20 | 18.04±0.13 |
| OSD | P<0.001, ↑ | 10.38±1.66 | 12.67±1.02 | 13.58±1.12 | 15.38±0.96 | 14.28±0.94 | 15.50±0.86 | 17.05±0.90 | 19.58±0.93 | 17.74±0.87 | 19.44±0.89 | 19.77±0.82 |
| **Liver disease** |  |  |  |  |  |  |  |  |  |  |  |  |
| Insomnia | P<0.001, ↑ | 0.79±0.10 | 0.86±0.10 | 0.99±0.10 | 0.96±0.08 | 0.94±0.08 | 1.17±0.08 | 1.43±0.09 | 1.63±0.09 | 1.69±0.09 | 2.00±0.09 | 2.08±0.07 |
| OSA | P<0.001, ↑ | . ± . | . ± . | . ± . | 1.35±0.17 | 1.28±0.07 | 1.37±0.06 | 1.86±0.08 | 1.91±0.07 | 2.03±0.08 | 2.26±0.06 | 2.44±0.05 |
| OSD | P<0.001, ↑ | 0.42±0.20 | 0.50±0.20 | 0.61±0.19 | 0.75±0.21 | 1.27±0.26 | 1.11±0.26 | 1.43±0.29 | 1.83±0.30 | 1.86±0.34 | 1.19±0.21 | 1.84±0.28 |
| **Lymphoma** |  |  |  |  |  |  |  |  |  |  |  |  |
| Insomnia | P>0.05 | 1.03±0.11 | 0.86±0.10 | 0.75±0.87 | 0.97±0.09 | 0.84±0.08 | 1.01±0.08 | 0.89±0.07 | 0.92±0.06 | 0.94±0.07 | 1.02±0.06 | 1.02±0.05 |
| OSA | P<0.001, ↑ | . ± . | . ± . | . ± . | 0.54±0.10 | 0.64±0.06 | 0.74±0.05 | 0.72±0.04 | 0.80±0.04 | 0.80±0.04 | 0.85±0.03 | 0.88±0.03 |
| OSD | P>0.05 | 0.76±0.29 | 0.80±0.25 | 0.42±0.15 | 0.67±0.21 | 0.78±0.22 | 0.71±0.21 | 0.73±0.21 | 0.84±0.22 | 0.80±0.20 | 0.86±0.19 | 0.88±0.19 |
| **Fluid/electrolyte** |  |  |  |  |  |  |  |  |  |  |  |  |
| Insomnia | P<0.001, ↑ | 18.11±0.56 | 19.06±0.50 | 20.17±0.54 | 22.59±0.49 | 23.29±0.46 | 23.60±0.44 | 25.50±0.47 | 25.57±0.42 | 26.74±0.46 | 27.13±0.41 | 27.26±0.27 |
| OSA | P<0.001, ↑ | . ± . | . ± . | . ± . | 18.38±0.61 | 19.84±0.50 | 20.27±0.40 | 22.01±0.47 | 22.00±0.38 | 23.14±0.38 | 24.15±0.34 | 24.66±0.19 |
| OSD | P<0.001, ↑ | 14.43±2.82 | 16.36±1.16 | 17.29±1.02 | 18.42±1.07 | 20.14±1.03 | 20.41±0.95 | 19.57±0.90 | 23.19±1.03 | 23.03±1.15 | 23.90±1.12 | 25.43±0.90 |
| **Metastatic cancer** |  |  |  |  |  |  |  |  |  |  |  |  |
| Insomnia | P<0.001, ↑ | 1.87±0.16 | 1.83±0.14 | 1.81±0.14 | 2.11±0.13 | 1.91±0.12 | 2.40±0.21 | 2.17±0.13 | 2.04±0.10 | 2.39±0.13 | 2.36±0.10 | 2.37±0.09 |
| OSA | P<0.001, ↑ | . ± . | . ± . | . ± . | 0.99±0.13 | 1.00±0.05 | 1.20±0.06 | 1.40±0.06 | 1.33±0.06 | 1.35±0.08 | 1.38±0.06 | 1.43±0.05 |
| OSD | P<0.01, ↑ | 1.00±0.30 | 1.14±0.31 | 0.93±0.23 | 1.56±0.31 | 1.37±0.29 | 1.43±0.30 | 1.58±0.28 | 2.28±0.33 | 1.66±0.26 | 2.06±0.32 | 1.61±0.25 |
| **Neurological disorders** |  |  |  |  |  |  |  |  |  |  |  |  |
| Insomnia | P<0.001, ↑ | 6.29±0.32 | 5.78±0.25 | 6.40±0.24 | 6.86±0.23 | 8.06±0.24 | 10.30±0.27 | 11.59±0.27 | 12.43±0.26 | 11.68±0.24 | 11.96±0.22 | 12.53±0.19 |
| OSA | P<0.001, ↑ | . ± . | . ± . | . ± . | 4.36±0.27 | 5.46±0.16 | 7.44±0.15 | 8.23±0.20 | 8.17±0.16 | 8.78±0.16 | 9.15±0.14 | 9.26±0.09 |
| OSD | P<0.001, ↑ | 6.65±1.32 | 7.80±0.82 | 8.14±0.76 | 9.13±0.82 | 10.18±0.78 | 14.06±0.85 | 13.89±0.91 | 14.25±0.80 | 14.72±0.81 | 15.60±0.79 | 15.15±0.72 |
| **Obesity** |  |  |  |  |  |  |  |  |  |  |  |  |
| Insomnia | P<0.001, ↑ | 4.46±0.36 | 4.50±0.27 | 4.95±0.24 | 5.17±0.23 | 5.82±0.32 | 6.46±0.26 | 7.40±0.31 | 8.11±0.25 | 8.56±0.31 | 9.24±0.24 | 9.89±0.18 |
| OSA | P<0.001, ↑ | . ± . | . ± . | . ± . | 31.90±0.81 | 33.50±0.75 | 34.77±0.62 | 38.06±0.70 | 38.04±0.62 | 38.72±0.61 | 40.50±0.47 | 42.02±0.26 |
| OSD | P<0.001, ↑ | 6.45±1.42 | 9.23±1.02 | 9.10±1.07 | 10.92±0.95 | 8.84±0.70 | 9.90±0.71 | 11.62±0.97 | 10.12±0.74 | 12.94±0.87 | 12.22±0.72 | 14.58±0.72 |
| **Paralysis** |  |  |  |  |  |  |  |  |  |  |  |  |
| Insomnia | P>0.05 | 3.77±0.30 | 2.60±0.24 | 2.71±0.24 | 2.62±0.22 | 2.58±0.19 | 2.53±0.16 | 2.62±0.17 | 2.76±0.17 | 2.41±0.13 | 2.63±0.14 | 2.74±0.10 |
| OSA | P<0.01, ↑ | . ± . | . ± . | . ± . | 1.59±0.20 | 1.77±0.10 | 1.65±0.08 | 1.93±0.09 | 1.81±0.06 | 2.01±0.07 | 1.82±0.05 | 2.03±0.04 |
| OSD | P>0.05 | 3.01±0.76 | 3.46±0.73 | 2.51±0.45 | 2.79±0.45 | 3.76±0.54 | 3.17±0.44 | 3.17±0.67 | 2.84±0.42 | 3.19±0.49 | 2.90±0.38 | 3.62±0.46 |
| **Peripheral vascular** |  |  |  |  |  |  |  |  |  |  |  |  |
| Insomnia | P<0.001, ↑ | 5.38±0.26 | 5.94±0.27 | 5.34±0.27 | 5.99±0.25 | 6.29±0.27 | 6.64±0.21 | 6.81±0.22 | 7.29±0.21 | 7.00±0.21 | 7.85±0.22 | 7.02±0.15 |
| OSA | P<0.001, ↑ | . ± . | . ± . | . ± . | 6.95±0.42 | 7.65±0.32 | 8.20±0.22 | 8.92±0.28 | 8.79±0.21 | 8.72±0.20 | 9.48±0.22 | 9.60±0.12 |
| OSD | P<0.001, ↑ | 4.58±1.02 | 5.21±0.68 | 5.75±0.74 | 5.79±0.60 | 6.89±0.67 | 6.90±0.60 | 8.08±0.70 | 7.32±0.63 | 6.02±0.57 | 7.97±0.57 | 7.43±0.56 |
| **Psychoses** |  |  |  |  |  |  |  |  |  |  |  |  |
| Insomnia | P<0.001, ↑ | 4.44±0.31 | 4.59±0.33 | 4.48±0.24 | 4.86±0.21 | 5.47±0.43 | 5.24±0.20 | 5.61±0.20 | 5.96±0.24 | 6.33±0.24 | 6.90±0.24 | 7.01±0.16 |
| OSA | P<0.001, ↑ | . ± . | . ± . | . ± . | 2.61±0.21 | 2.60±0.13 | 2.84±0.10 | 3.29±0.12 | 3.21±0.10 | 3.64±0.14 | 3.74±0.11 | 3.82±0.08 |
| OSD | P<0.001, ↑ | 3.94±0.92 | 3.90±0.59 | 5.11±1.28 | 4.54±0.54 | 6.62±0.67 | 6.43±0.68 | 8.00±0.73 | 5.39±0.54 | 7.61±0.65 | 7.48±0.73 | 7.67±0.60 |
| **Pulmonary circ.** |  |  |  |  |  |  |  |  |  |  |  |  |
| Insomnia | P<0.001, ↑ | 0.80±0.12 | 1.08±0.13 | 1.00±0.10 | 1.15±0.10 | 1.09±0.08 | 1.62±0.10 | 1.89±0.10 | 2.30±0.10 | 2.22±0.11 | 2.30±0.10 | 2.19±0.08 |
| OSA | P<0.001, ↑ | . ± . | . ± . | . ± . | 4.72±0.29 | 5.10±0.21 | 5.65±0.16 | 6.35±0.21 | 6.07±0.15 | 6.22±0.16 | 6.60±0.15 | 6.19±0.09 |
| OSD | P<0.001, ↑ | 1.35±0.42 | 1.58±0.34 | 1.20±0.28 | 2.48±0.54 | 1.78±0.33 | 1.78±0.32 | 2.65±0.46 | 2.23±0.31 | 2.93±0.40 | 2.74±0.33 | 2.81±0.34 |
| **Renal failure** |  |  |  |  |  |  |  |  |  |  |  |  |
| Insomnia | P<0.001, ↑ | 3.02±0.19 | 3.28±0.24 | 3.44±0.20 | 4.23±0.18 | 7.50±0.28 | 8.67±0.25 | 9.95±0.29 | 10.78±0.27 | 11.71±0.30 | 12.20±0.26 | 12.23±0.20 |
| OSA | P<0.001, ↑ | . ± . | . ± . | . ± . | 14.70±0.63 | 16.27±0.43 | 18.30±0.36 | 19.48±0.43 | 20.28±0.39 | 21.88±0.40 | 23.30±0.32 | 23.12±0.18 |
| OSD | P<0.001, ↑ | 3.08±0.76 | 2.94±0.52 | 3.90±0.49 | 4.74±0.53 | 7.48±1.16 | 9.25±0.77 | 8.87±0.82 | 8.76±0.65 | 10.88±0.77 | 11.84±0.67 | 11.89±0.67 |
| **Non-metastatic cancer** |  |  |  |  |  |  |  |  |  |  |  |  |
| Insomnia | P<0.001, ↓ | 9.43±0.40 | 2.33±0.16 | 2.29±0.14 | 2.53±0.13 | 2.51±0.13 | 2.42±0.11 | 2.79±0.13 | 2.62±0.10 | 2.50±0.11 | 2.71±0.09 | 2.72±0.08 |
| OSA | P<0.001, ↑ | . ± . | . ± . | . ± . | 1.82±0.15 | 1.69±0.07 | 1.88±0.06 | 1.97±0.07 | 1.96±0.07 | 2.04±0.08 | 2.14±0.06 | 2.11±0.04 |
| OSD | P<0.001, ↓ | 6.31±1.31 | 2.40±0.45 | 2.09±0.38 | 2.41±0.37 | 2.87±0.42 | 2.17±0.35 | 1.85±0.29 | 2.10±0.31 | 2.38±0.34 | 2.23±0.28 | 1.81±0.29 |
| **Peptic ulcer** |  |  |  |  |  |  |  |  |  |  |  |  |
| Insomnia | P<0.001, ↓ | 2.67±0.18 | 0.17±0.05 | 0.11±0.03 | 0.08±0.02 | 0.04±0.02 | 0.11±0.02 | 0.04±0.01 | 0.05±0.01 | 0.03±0.01 | 0.07±0.01 | 0.04±0.01 |
| OSA | P>0.05 | . ± . | . ± . | . ± . | 0.03±0.02 | 0.02±0.08 | 0.04±0.01 | 0.03±0.01 | 0.03±0.01 | 0.03±0.01 | 0.03±0.01 | 0.03±0.00 |
| OSD | P<0.01, ↓ | 1.37±0.38 | 0.09±0.09 | 0.07±0.07 | 0.11±0.08 | . ± . | 0.05±0.05 | 0.16±0.09 | . ± . | 0.09±0.06 | . ± . | 0.16±0.08 |
| **Valvular disease** |  |  |  |  |  |  |  |  |  |  |  |  |
| Insomnia | P<0.001, ↑ | 3.84±0.25 | 4.13±0.22 | 3.82±0.21 | 4.41±0.23 | 4.69±0.20 | 5.06±0.19 | 5.31±0.21 | 5.21±0.17 | 5.19±0.19 | 5.14±0.16 | 4.97±0.13 |
| OSA | P<0.001, ↑ | . ± . | . ± . | . ± . | 4.79±0.32 | 5.20±0.20 | 5.41±0.16 | 5.60±0.18 | 5.35±0.14 | 5.50±0.15 | 5.79±0.13 | 5.86±0.09 |
| OSD | P<0.05, ↑ | 2.78±0.69 | 5.35±0.71 | 5.11±0.55 | 4.60±0.58 | 5.08±0.55 | 4.59±0.49 | 5.14±0.51 | 4.58±0.43 | 4.81±0.51 | 5.65±0.49 | 5.66±0.49 |
| **Weight loss** |  |  |  |  |  |  |  |  |  |  |  |  |
| Insomnia | P<0.001, ↑ | 2.32±0.31 | 2.95±0.22 | 2.70±0.18 | 2.88±0.18 | 3.33±0.26 | 3.75±0.16 | 4.25±0.19 | 4.53±0.19 | 4.42±0.17 | 5.56±0.25 | 5.27±0.14 |
| OSA | P<0.001, ↑ | . ± . | . ± . | . ± . | 1.05±0.14 | 1.27±0.13 | 1.29±0.08 | 1.90±0.13 | 1.97±0.12 | 2.08±0.11 | 2.72±0.14 | 2.57±0.07 |
| OSD | P<0.001, ↑ | 0.75±0.28 | 1.46±0.33 | 3.03±0.50 | 2.98±0.44 | 2.30±0.37 | 3.39±0.46 | 4.33±0.62 | 4.46±0.54 | 4.43±0.51 | 5.07±0.46 | 5.38±0.46 |
|  |  |  |  |  |  |  |  |  |  |  |  |  |
| **Total co-morbidities, Mean±SE** |  |  |  |  |  |  |  |  |  |  |  |  |
| Insomnia | P<0.05, ↑ | 2.28±0.03 | 2.28±0.03 | 2.34±0.03 | 2.48±0.03 | 2.62±0.03 | 2.74±0.02 | 2.87±0.03 | 2.97±0.02 | 3.00±0.02 | 3.09±0.02 | 3.10±0.01 |
| OSA | P<0.01, ↑ | . ± . | . ± . | . ± . | 3.11±0.04 | 3.29±0.04 | 3.42±0.04 | 3.60±0.04 | 3.60±0.03 | 3.70±0.03 | 3.85±0.02 | 3.87±0.01 |
| OSD | P<0.05, ↑ | 1.92±0.24 | 2.22±0.05 | 2.31±0.05 | 2.46±0.05 | 2.62±0.06 | 2.77±0.05 | 2.88±0.05 | 2.89±0.04 | 3.06±0.05 | 3.12±0.05 | 3.19±0.04 |
| **Unwt. N**** |  |  |  |  |  |  |  |  |  |  |  |  |
| Insomnia |  | 8,452 | 9,587 | 11,884 | 15,812 | 18,258 | 22,999 | 21,695 | 31,229 | 28,486 | 41,033 | 39,430 |
| OSA |  | … | … | … | 6,658 | 45,082 | 65,624 | 68,933 | 106,022 | 106,502 | 151,771 | 152,299 |
| OSD |  | 1,259 | 1,278 | 1,576 | 1,643 | 1,771 | 1,924 | 1,890 | 2,153 | 2,056 | 2,675 | 2,489 |
| **Wt. N**** |  |  |  |  |  |  |  |  |  |  |  |  |
| Insomnia |  | 39,029 | 45,212 | 56,280 | 75,299 | 86,524 | 109,452 | 102,490 | 152,628 | 138,020 | 192,972 | 197,150 |
| OSA |  | … | … | … | 31,747 | 214,580 | 313,351 | 325,358 | 516,652 | 516,769 | 706,038 | 761,495 |
| OSD |  | 5,633 | 6,007 | 7,514 | 7,808 | 8,402 | 9,126 | 8,865 | 10,435 | 9,925 | 12,657 | 12,445 |

*Abbreviations*: AIDS=Acquired Immune deficiency Syndrome; CHF=Congestive Heart Failure; LOS=Length of stay; MR=mortality Risk; NIS=Nationwide Inpatient Sample; OSA=Obstructive Sleep Apnea; OSD=Other Sleep Disturbances; TC=Total Charges; Unwt=Unweighted; Wt=Weighted.

*Descending trend; ** For sample with complete data on all co-morbidities (i.e. total co-morbidities), 2002-2012

**Table S4.** Trends in MR (%), LOS(days) and TC($) among patients with insomnia, OSA and OSD; NIS, 2002 to 2012

|  | **2002** | **2003** | **2004** | **2005** | **2006** | **2007** | **2008** |
| --- | --- | --- | --- | --- | --- | --- | --- |
| **Unwt. N**† |  |  |  |  |  |  |  |
| Insomnia | 8,660 | 9,812 | 11,875 | 15,800 | 18,244 | 22,988 | 21,674 |
| OSA | .. | .. | .. | 6,654 | 45,062 | 65,612 | 68,875 |
| OSD | 1,277 | 1,322 | 1,574 | 1,641 | 1,771 | 1,923 | 1,889 |
| **Wt. N**† |  |  |  |  |  |  |  |
| Insomnia | 40,087 | 46,277 | 56,236 | 75,236 | 86,458 | 109,391 | 102,391 |
| OSA | .. | .. | .. | 31,727 | 214,479 | 313,288 | 325,081 |
| OSD | 5,734 | 6,217 | 7,504 | 7,800 | 8,402 | 9,120 | 8,860 |
|  |  |  |  |  |  |  |  |
| **MR, %±SE** |  |  |  |  |  |  |  |
| Insomnia P<0.01, ↓ | 1.47±0.14 | 1.30±0.12 | 1.20±0.12 | 1.16±0.09 | 1.08±0.09 | 1.12±0.08 | 1.27±0.10 |
| OSA P<0.001, ↓ | . ± . | . ± . | . ± . | 1.67±0.17 | 1.77±0.08 | 1.72±0.06 | 1.81±0.07 |
| OSD P>0.05 | 1.41±0.43 | 1.68±0.41 | 1.10±0.28 | 0.81±0.26 | 0.80±0.21 | 0.91±0.22 | 1.24±0.23 |
| **LOS(days), Mean±SE** |  |  |  |  |  |  |  |
| Insomnia P<0.001, ↓ | 6.14±0.19 | 5.81±0.12 | 5.94±0.20 | 5.81±0.19 | 5.62±0.13 | 5.45±0.09 | 5.41±0.10 |
| OSA P<0.01, ↓ | . ± . | . ± . | . ± . | 5.25±0.09 | 5.38±0.07 | 5.20±0.06 | 5.26±0.06 |
| OSD P<0.001, ↓ | 5.29±0.31 | 5.21±0.21 | 5.60±0.28 | 5.84±0.34 | 5.33±0.19 | 5.06±0.16 | 5.27±0.24 |
| **TC($), Mean±SE** |  |  |  |  |  |  |  |
| Insomnia P<0.05, ↑ | 22249.66±817.12 | 24219.39±799.59 | 25762.58±876.19 | 25657.62±826.70 | 25889.92±663.11 | 29752.56±747.84 | 31527.54±975.95 |
| OSA P<0.01, ↑ | . ± . | . ± . | . ± . | 37560.56±1419.44 | 37768.81±1028.74 | 38541.53±1029.99 | 42093.33±1124.62 |
| OSD P<0.05, ↑ | 18263.55±2096.23 | 22675.00±1070.44 | 24198.64±1044.07 | 26384.24±1409.11 | 25860.63±1088.51 | 26257.61±1044.31 | 29896.50±1208.27 |

|  |  | **2009** | **2010** | **2011** | **2012** | **β*±SE** | **β**±SE** |
| --- | --- | --- | --- | --- | --- | --- | --- |
| **Unwt. N**† |  |  |  |  |  |  |  |
| Insomnia |  | 31,223 | 28,475 | 40,990 | 39,417 |  |  |
| OSA |  | 105,995 | 106,489 | 151,506 | 152,281 |  |  |
| OSD |  | 2,153 | 2,055 | 2,674 | 2,489 |  |  |
| **Wt. N**† |  |  |  |  |  |  |  |
| Insomnia |  | 152,599 | 137,968 | 192,776 | 197,085 |  |  |
| OSA |  | 516,528 | 516,707 | 704,855 | 761,405 |  |  |
| OSD |  | 10,435 | 9,921 | 12,653 | 12,445 |  |  |
|  |  |  |  |  |  |  |  |
| **MR, %±SE** |  |  |  |  |  |  |  |
| Insomnia |  | 1.19±0.08 | 1.27±0.08 | 1.09±0.06 | 0.98±0.05 | **-0.02±0.01##** | **-0.04±0.01###** |
| OSA |  | 1.78±0.06 | 1.61±0.06 | 1.55±0.04 | 1.57±0.04 | **-0.03±0.01###** | **-0.06±0.01###** |
| OSD |  | 1.32±0.25 | 0.70±0.19 | 0.81±0.18 | 1.04±0.20 | -0.04±0.03 | **-0.07±0.03#** |
| **LOS(days), Mean±SE** |  |  |  |  |  |  |  |
| Insomnia |  | 5.66±0.14 | 5.32±0.11 | 5.41±0.11 | 5.35±0.06 | **-0.06±0.01###** | **-0.10±0.01###** |
| OSA |  | 5.07±0.05 | 4.96±0.05 | 4.90±0.04 | 4.88±0.03 | **-0.08±0.01###** | **-0.13±0.01###** |
| OSD |  | 5.38±0.28 | 5.55±0.30 | 5.21±0.16 | 5.39±0.14 | -0.01±0.02 | **-0.05±0.02#** |
| **TC($), Mean±SE** |  |  |  |  |  |  |  |
| Insomnia |  | 33725.92±822.22 | 35904.99±982.74 | 37029.55±894.24 | 38177.33±543.18 | **+1722.40±87.61###** | **+1491.87±87.52###** |
| OSA |  | 43606.15±1096.11 | 43525.17±1207.52 | 45525.09±1271.66 | 46518.40±626.97 | **+1414.03±189.43###** | **+1235.29±191.18###** |
| OSD |  | 31495.19±928.56 | 32584.02±1210.63 | 36306.22±2107.59 | 35449.86±939.67 | **+1635.19±156.46###** | **+1450.38±150.95###** |

*Abbreviations*: LOS=Length of stay; MR=mortality Risk; NIS=Nationwide Inpatient Sample; OSA=Obstructive Sleep Apnea; OSD=Other Sleep Disturbances; TC=Total Charges; Unwt=Unweighted; Wt=Weighted.

* β=Log(odds ratio) from logit model in the case of mortality status and linear regression coefficient from linear regression model for TC and LOS with year as the only covariate.

** β=Log(odds ratio) from logit model in the case of mortality status and linear regression coefficient from linear regression model for TC and LOS with year as the main covariate. Control was additionally made on the following covariates: age, sex and total number of co-morbidities.

† Sample of older adults with sleep disturbances having complete data on mortality status. Other sample sizes were comparable per year.

#P-trend<0.05; ##P-trend<0.01; ### P-trend<0.001.
